# Insights on the sex determination, vector capacity and ecological biology from a chromosomal level genome of vector mosquito, *Armigeres subulbatus*

**DOI:** 10.1186/s40249-025-01353-1

**Published:** 2025-08-08

**Authors:** Peiwen Liu, Feng Liu, Hao-Ran Lu, Jinbao Gu, Xiaohong Zhou, Yang Wu, Zhen Zou, Xiang Guo, Wenqiang Yang, Shan Li, Ziyao Li, Xiao-Guang Chen

**Affiliations:** 1 Department of Pathogen Biology, Institute of Tropical Medicine, School of Public Health, Southern Medical University, Guangzhou, 510515 Guangdong China; 2 Institute of Public Health, Guangzhou Medical University & Guangzhou Center for Disease Control and Prevention (Guangzhou Health Supervision institute), Guangzhou, 510440 People’s Republic of China; 3 Institute of Infectious Diseases, Shenzhen Bay Laboratory, Shenzhen, 518107 China; 4 State Key Laboratory of Integrated Management of Pest Insects and Rodents, Institute of Zoology, Chinese Academy of Sciences, Beijing, 100101 China; 5 University of Chinese Academy of Sciences, Beijing, 100101 China; 6 Guagnzhou Customs Technology Center, Guangzhou, 510000 China; 7 Guangdong Provincial Key Laboratory of Infection Immunity and Inflammation, Shenzhen, 518107 China

**Keywords:** *Armigeres subalbatus*, Genome assembly, Vector biology, Male determination, Diapause, Immune system

## Abstract

**Background:**

Mosquitoes with aggressive biting behavior are important disease vectors threatening public health. *Armigeres subalbatus*, as an emerging arbovirus and filarial disease vector, exhibits aggressive host-seeking behavior and unique breeding preference for contaminated water. However, the molecular mechanisms underlying these biological characteristics remain poorly understood. This study aimed to generate a high-quality genome assembly and characterize the genetic basis of vector competence and environmental adaptation in *Ar. subalbatus*.

**Methods:**

We sequenced and assembled the *Ar. subalbatus* genome using Oxford Nanopore long-read sequencing, Illumina short-read sequencing, and Hi-C technology. Comparative genomic analysis was performed to identify gene families related to detoxification, diapause, innate immunity, and sex determination. Gene structure analysis focused on the male-determining factor and its evolutionary relationships with other mosquito vectors.

**Results:**

The genome assembly consists of three chromosomes, with a total size of 1.33 Gbp and an N50 of 430.15 Mbp (GenBank assembly: GCA_024139115.2), displaying 99.4% Benchmarking Universal Single-Copy Orthologs (BUSCO) completeness. We identified the gene structure of the male-determining factor (*AsuMf*) and characterized its evolutionary relationship with other mosquito vectors. The analysis revealed expanded detoxification-related gene families including cytochrome P450s, which may facilitate adaptation to contaminated breeding sites. We characterized 566 putative diapause-related genes that could potentially contribute to geographical expansion, 334 innate immune genes, and 1673 endogenous viral elements, indicating complex virus-host interactions throughout evolution.

**Conclusions:**

Our study provides insights into the molecular basis of vector competence and adaptation in *Ar. subalbatus*. The expanded detoxification gene families may enable the species to survive in polluted environments, while the identified diapause-related genes could explain its geographical expansion capabilities. These findings establish a foundation for developing novel vector control strategies targeting this emerging disease vector.

**Graphical Abstract:**

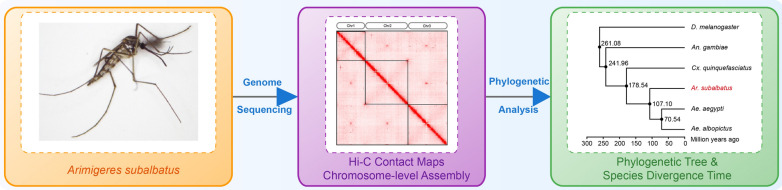

**Supplementary Information:**

The online version contains supplementary material available at 10.1186/s40249-025-01353-1.

## Background

*Armigeres subalbatus*, a significant vector mosquito species positioned taxonomically between *Aedes* and *Culex* within Culicinae, has emerged as a growing public health concern due to its aggressive nature and unique feeding habits [1, 2]. This species transmits various pathogens, including filarial worms (*Brugia pahangi* and *B. malayi*) and Japanese encephalitis virus [3, 4]. Recent studies have expanded our understanding of its vector potential, with isolations of Zika virus from wild *Ar. subalbatus* populations and experimental susceptibility studies indicating its possible role in Zika virus transmission [5–8]. Notably, the species' preference for breeding in contaminated water sources aligns with the urine-water-mosquito-human transmission cycle of Zika virus [9], raising additional concerns about its potential to facilitate viral spread. Furthermore, climate change has driven the northward expansion of this mosquito species [10], potentially extending the geographical range of mosquito-borne diseases into previously unaffected regions.

Sex determination mechanisms in mosquitoes represent a striking example of rapid molecular evolution, with diverse strategies emerging across species [11]. Several male-determining factors have been identified: the Y-linked genes *Yob* in *Anopheles gambiae* [12] and *Guy1* in *An. stephensi* [13], the chromosome 1-linked gene *Nix* in both *Aedes aegypti* [14, 15] and *Ae. albopictus* [16, 17], and an uncharacterized M factor on chromosome 1 in *Culex quinquefasciatus*. Recently, *AsuMf*, a DBHS gene on chromosome 3, was identified as the male-determining factor in *Ar. subalbatus* [18], suggesting a distinct evolutionary path that warrants further investigation. Vector competence in mosquitoes is fundamentally shaped by their immune and chemosensory systems, which influence both pathogen transmission and ecological adaptation [19–21]. While mosquito species show marked differences in immune responses and chemosensory gene repertoires, the relationship between these variations and vector capabilities remains poorly understood [22]. Endogenous viral elements (EVEs), inherited viral sequence integrations in mosquito genomes, contribute to antiviral immunity through mechanisms including piRNA production [23, 24]. Diapause capability, essential for survival in adverse conditions, also varies among mosquito species [25, 26]. Despite well-documented egg diapause in *Aedes* and adult diapause in *Culex* mosquito, the molecular basis of larval diapause in *Ar. subalbatus* remains poorly understood [25–27]. *Ar. subalbatus* represents an intriguing model, exhibiting distinctive pathogen susceptibility patterns and host-seeking behaviors [8, 28, 29]. Given its expanding geographical distribution and potential responses to climate change [10], a high-quality genome assembly is urgently needed to understand these molecular mechanisms and adaptations.

To address the lack of genomic data for this overlooked vector species, we have assembled a chromosome-level genome of *Ar. subalbatus* using a combination of next generation sequencing (NGS), Oxford Nanopore Technologies (ONT) sequencing, and Hi-C technologies. This highly contiguous and well-annotated genome provides a foundation for investigating the species' distinctive biological features, including its sex determination mechanism, immunological responses, chemosensory capabilities, and diapause regulation. Through comprehensive comparative genomic analyses, we reveal unique aspects of genome organization, gene family evolution, and endogenous viral elements that may underline the species' adaptive capabilities and vector competence. Our findings not only advance our understanding of mosquito genome evolution but also provide valuable resources for developing novel vector control strategies in response to the expanding range and emerging disease threats posed by this important vector species.

## Methods

### Mosquito maintenance and DNA preparation

*Armigeres subalbatus* specimens were collected on Aug 30, 2018 from a campus (23°12′03.1′′N, 113°17′20.1′′E) in Guangzhou City, Guangdong Province, China, and subsequently maintained in a laboratory setting [8]. The mosquitoes were kept under standard insectary conditions at a temperature of 27 ± 1 °C, relative humidity of 70–80%, and a 16-h light and 8-h dark cycle. Larvae were reared in stainless steel trays (24 cm × 34 cm × 6 cm) and fed daily with turtle food (INCH-GOLD^®^, Huizhou City, China). Pupae were transferred with a pipette into 300-ml plastic cups containing dechlorinated water, and then moved to mosquito cages (25 cm × 25 cm × 35 cm). Adults were fed with 10% glucose water. We performed a single pair cross between a male and a female individual; from the progeny of this cross, we randomly picked a male and a female and made them mate. We repeated this procedure for six generations, after which we let the progeny of a single-pair mating interbreed. Pupae were individually put into EP tube to separate male and female. Genomic high-molecular weight (HMW) DNA was isolated using the QiaAMP DNA micro kit (Qiagen, Germantown, MD, USA).

### Genome sequencing and assembly

#### Sample collection and DNA extraction

Adult male and female mosquitoes were collected post eclosion. The specimens were immediately flash-frozen in liquid nitrogen and stored at −80 °C until DNA extraction. Genomic DNA was extracted using the DNeasy Blood & Tissue Kit (Qiagen, Hilden, Germany) following the manufacturer’s protocol. DNA quality and quantity were assessed using a NanoDrop spectrophotometer (Thermo Fisher Scientific, Waltham, MA, USA), 0.5% agarose gel electrophoresis, and a Qubit 2.0 fluorometer (Invitrogen, Carlsbad, CA, USA), respectively.

#### Oxford nanopore technologies (ONT) sequencing

High molecular weight DNA was prepared for long-read sequencing using the Ligation Sequencing Kit (SQK-LSK109, Oxford Nanopore Technologies, Oxford, UK). Approximately 1 μg of DNA was used for library preparation. The DNA was repaired and end-prepped using the NEBNext Ultra II End Repair/dA-Tailing Module (New England Biolabs, Ipswich, MA, USA). Adapter ligation was performed using the Adapter Mix provided in the SQK-LSK109 ligation sequencing kit (Oxford Nanopore Technologies, Oxford, UK). The prepared library was loaded onto a PromethION Flow Cell (R9.4.1, Oxford Nanopore Technologies) and sequenced on a PromethION device (Oxford Nanopore Technologies) for 72 h. Basecalling was performed using Guppy (v3.6.0, Oxford Nanopore Technologies) with the default filtering setting of Qscore > 7 [30], generating a total of 160 Gb of ONT long-read data that provided approximately 120 × coverage depth.

#### De novo genome assembly

About 6.9 million long reads with 15.9 billion base-pair generated from ONT sequencing were assembled de novo using NextDenovo (v2.4.0) [31]. The configuration parameters for NextDenovo were set to optimize for the expected genome size to 1 gigabase. The assembly process involved several steps: error correction, assembly, and consensus calling. The initial genome assembly was polished with ONT reads using NextPolish (v1.4.1) for four rounds to correct sequencing errors [32].

#### Short-read sequencing and genome polishing

Approximately 300 ng of genomic DNA was used to prepare DNA sequencing libraries for each parent following using the NEBNext Ultra II FS DNA Library Prep Kit for Illumina (New England Biolabs, Ipswich, MA, USA). The libraries were performed 150 bp paired-end sequencing separately using Illumina HiSeq 4000 (100 × each) according to the manufacturer's instructions. Yielding 283.33 million female and 282.62 million male reads were used to polish the ONT assembly with NextPolish (v1.4.1) for three rounds, improving the base-level accuracy of the genome assembly.

#### Removal of redundancies

Following polishing, the genome assembly was further processed to remove redundant sequences using Purge_Dups (v1.2.5) [33]. The pipeline involves mapping Illumina short reads to the assembly to identify and remove duplicated regions, thereby improving the contiguity and accuracy of the final assembly.

#### Hi-C sequencing and chromosome scaffolding

For chromosome-level scaffolding, Hi-C libraries were prepared using the Proximo Hi-C kit (Phase Genomics, Seattle, WA, USA) according to the manufacturer’s instructions.

Thirty male pupae were collected and fixed with paraformaldehyde-based buffer and homogenized with tissue homogenizer, followed by cell lysis. The chromatin was digested with MboI restriction enzyme (New England Biolabs, Ipswich, MA, USA), and the sticky ends were biotinylated and proximity-ligated to form chimeric junctions. The DNA was purified and sheared to a size of 300–500 bp, and biotinylated fragments were pulled down with streptavidin beads. The Hi-C libraries were then prepared using standard Illumina library preparation protocols and sequenced on an Illumina NovaSeq 6000 platform (2 × 150 bp), generating a total of 274 Gb of Hi-C data that provided approximately 210 × coverage depth. Hi-C data were processed using Juicer (v1.6) and 3D-DNA (v180922) pipelines [34, 35]. The draft genome assembly was scaffolded into chromosome-level assemblies using Hi-C contact maps according to their 3-dimensional contacts. Multiple misassemblies were detected and fixed.

#### Quality assessment

The completeness and quality of the final genome assembly were evaluated using a faster and more accurate reimplementation of BUSCO (Benchmarking Universal Single-Copy Orthologs, v4.1.4), the compleasem tool with the diptera_odb10 database [36, 37]. Genome assembly metrics, including N50, total length, and GC content, were calculated using QUAST (v5.0.2) [38].

### Genome annotation

The assembled genome was submitted to the National Center for Biotechnology Information (NCBI) under the accession number GCA_024139115.2 and annotated using NCBI’s Eukaryotic Genome Annotation Pipeline. The annotation, referred to as NCBI *Ar. subalbatus* Annotation Release GCF_024139115.2-RS_2024_02, includes predicted gene models for protein-coding and non-coding genes and pseudogenes and is available from NCBI’s genome FTP site and web resources. Models were predicted using NCBI’s Gnomon algorithm using alignments of transcripts, proteins, and RNA-seq data as evidence. The evidence datasets used for Release GCF_024139115.2-RS_2024_02 included alignments of available *Ar. subalbatus* mRNAs and expressed sequence tags (ESTs), 1.87 billion RNA-seq reads from 31 sequence read archive (SRA) runs from a wide range of samples, and RefSeq proteins from *Ae. aegypti* and other insects. The quality of the annotation was evaluated using BUSCO v4.1.4 with the diptera_odb10 database [37, 39]. The complete genome assembly and annotation workflow is summarized in Additional file 1: Figure S1.

### Genomic and transcriptomic reads alignment and quality assessment

Raw DNA-seq and RNA-seq data (BioProject PRJNA834573) were processed through distinct alignment pipelines. For DNA-seq reads, BWA-MEM (v0.7.17) [40] was employed using the following parameters: maximum insert size of 1000 bp (-I 1000), gap opening penalty score of 6 (-O 6), and minimum seed length of 19 bp (-k 19). For RNA-seq data, HISAT2 (v2.2.1) [41] was used with specific spliced alignment parameters: maximum intron length of 500,000 bp (–max-intronlen 500,000), allowing up to 2 mismatches per seed (-N 2), and with novel splice site detection enabled (–novel-splicesite-outfile). The alignment outputs from both pipelines were processed using SAMtools (v1.9) [42] for format conversion, sorting, and indexing, with mapping statistics generated using bamtools (v2.5.2) [43].

To comprehensively assess RNA-seq alignment quality and splicing patterns, we further analyzed the STAR aligner (v2.7.10a) [44] output files. Four key metrics were quantified for each sample: uniquely mapped reads (percentage of reads mapping to a single genomic location), multiply mapped reads (percentage of reads aligning to multiple loci), total spliced alignments (millions of reads spanning splice junctions), and annotated spliced alignments (percentage of spliced reads corresponding to annotated splice sites).

### Phylogenetic reconstruction and divergence time estimation

To reconstruct the phylogeny of mosquito species including *Ar. subalbatus*, we utilized genome assemblies of *An. gambiae*, *Ae. aegypti*, *Cx. quinquefasciatus*, and *D. melanogaster* as an outgroup. Each assembly, including that of *Ar. subalbatus*, was analyzed using BUSCO v4.1.4 [39] to identify universal single-copy orthologs from the OrthoDB dipteria_odb10 database [37]. Multiple sequence alignments of the orthologous proteins were performed using MAFFT v7.471 [45], followed by alignment trimming with trimAl v1.4 [4] (-gt 0.5 option) [46]. The resulting alignment was used to estimate the maximum likelihood species phylogeny using RAxML v8.0 [47] with the PROTGAMMAJTT model, which was chosen as it is a standard model for protein sequence phylogenetic analysis that incorporates the Jones-Taylor-Thornton substitution matrix and accounts for rate heterogeneity among sites using a gamma distribution, rooted with *D. melanogaster*. To estimate divergence times, we employed MCMCTree in the PAML v4.9 package [48] to calibrate branch lengths in terms of millions of years. We used two calibration points: 260 million years ago (MYA) for the common ancestor of mosquitoes and *D. melanogaster*, and 70 MYA for the divergence between *Ae. aegypti* and *Ae. albopictus*, based on previous molecular clock analyses [49].

### Identification of the M locus

Our previous study showed that the male-determining factor *AsuMf* is located on the chromosome 3 [18]. To overcome the challenges of repetitive hits, the sequences of chromosome 3 were twice repeat-masked against a combined repeat library from Diptera using RepeatMasker [50]. The chromosome quotient (CQ) was calculated for each 500-bp window across chromosome 3. The Chromosome Quotient (CQ) for a given 500-bp sequence window (*S*) was calculated using the following formula:

$${CQ}_{S}=\frac{{F}_{S}}{({M}_{S}+0.01)}$$

where F_*S*_ is the number of female Illumina reads aligned to *S*, and M_*S*_ is the number of male Illumina reads aligned to *S*. Normalization was not necessary for these datasets because the mean and median CQs of the autosomes (chromosome 1 and chromosome 2) are all near 1. A CQ value lower than 0.05 indicates that the sequences within the corresponding 500-bp window had at least 20-fold more hits to the male Illumina data than to the female Illumina data. Not every 500-bp window produces a CQ value because many were completely masked by RepeatMasker. To ensure that each CQ value represents a meaningful data point obtained with sufficient alignments, only sequences with more than 20 male hits were included in the calculation. The CQ values were then plotted against the chromosome location of the 500-bp window.

### Annotation and phylogenetic analysis of chemosensation and xenobiotic detoxification genes

Using Blast + v2.14.0 [51], the protein sequence of *Ar. subalbatus* was compared with previously reported chemosensation and xenobiotic detoxification genes from various haematophagous insects, including *An. gambiae*, *Ae. aegypti*, *Glossina morsitans*, *Pediculus humanus*, *Cimex lectularius* and *Ctenocephalides felis* [52–57], with an e-value threshold set at < 1e-5. The Interpro v101.0 [58] was utilized to annotate the domains of candidate chemosensation and xenobiotic detoxification genes identified in *Ar. subalbatus*. Subsequently, protein sequences were gathered for multiple sequence alignment using mafft v7.520 [59] with poorly aligned regions removed through trimAl using the “automated” option [46], followed by the construction of a maximum likelihood phylogenetic tree using FastTree v2.1.11 [60]. The Interactive Tree of Life (iTOL v6) was adopted for visualisation and annotation of the trees [61].

### Annotation and phylogenetic analysis of diapause-related genes

Our analysis delves into genome regions that have been demonstrated involving in the diapause program in *Ae. albopictus* populations, encompassing both temperate and subtropical habitats, and fully capable of undergoing diapause. This endeavor builds upon a robust foundation of previously published studies and our ongoing multi-omics investigations, while also integrating insights from diapause-related research conducted on other insect species [62–64]. First, to streamline the annotation process, we systematically established a comprehensive database of putative insect diapause-related genes extracted from both published literature and our ongoing analyses. Subsequently, the database underwent rigorous categorization into nine distinct gene sets, each representing pivotal putative diapause-related gene cluster. Among these, two gene sets stand out as sensory gene clusters, specifically focusing on phototransduction mechanisms and the transient receptor potential (TRP) channel family for mediating sensory perceptions pertinent to insect diapause. Furthermore, the remaining seven gene sets encompass gene clusters related to circadian rhythm regulation, hormone modulation, detoxification pathways, neuropeptide signaling, cold tolerance mechanisms, drought resistance strategies, and energy metabolism processes, all of which are intricately involved and vital components of the complex diapause machinery in insects [63].

Thereby, leveraging the aforementioned database of putative diapause-associated genes, we conducted a comparative genomic analysis of the characteristics of diapause-related genes in *Ar. subalbatus* against those identified in a non-diapause mosquito species, *Ae. aegypti* (GCF_002204515.2), as well as in other insect species that exhibit diverse diapause stages, including *Ae. albopictus* (GCF_035046485.1), *Cx. pipens* (GCF_16801865.2)*, An. gambiae* (GCF_943734735.2), and *D. melanogaster* (GCF_000001215.4). To achieve this, we mined the reference genome annotations of these five species for the presence of diapause genes, meticulously tallying their respective loci counts. Furthermore, among these putative diapause-related genes, we selected and employed the protein sequences of key representative genes including *Tret*, *lipase3*, and *cry,* to conduct phylogenetic analyses, aiming to elucidate their evolutionary relationships and potential functional conservation across species. Additionally, we predicted the transmembrane domains of TRETs utilizing the TMHMM-2.0 tool [65], which provides insights into their potential membrane-spanning domains, and thus their potential roles in cellular signaling or transport processes that may be crucial for adaptation and regulation of diapause.

### Annotation and phylogenetic analysis of innate immune genes

Using Blast + v2.12.0 + [51], the protein sequence of *Ar. subalbatus* was compared with previously reported innate immune genes from various organisms, including *Drosophila melanogaster*, *Anopheles gambiae*, *Aedes aegypti*, *Bombyx mori*, *Apis mellifera*, *Manduca sexta* and *Tribolium castaneum* [52–57], with an e-value threshold set at < 1e-5. The Interpro v99.0 [58] was utilized to annotate the domains of candidate immune genes identified in *Ar. subalbatus*. Additionally, predictions from DeepTMHMM [66] and SignalP [67] were integrated to identify transmembrane regions and signal peptides.

Once the innate immune genes of *Ar. subalbatus* were identified, they were categorized into four clusters: recognition, modulation, signaling, and effectors. Subsequently, protein sequences were gathered for multiple sequence alignment using mafft [59], followed by the construction of a maximum likelihood phylogenetic tree using IQ-TREE v2.3.0 [60], with 1000 bootstrap resamples.

### Identification of endogenous viral elements

EVEs in the *Ar. subalbatus* genome (GCA_024139115.2) were identified using the Refine_EVEs_annotation pipeline (https://github.com/BonizzoniLab/Refine_EVEs_annotation), a BLAST-based method [68]. The process utilized a curated database of 683,242 viral proteins from NCBI RefSeq as of May 2024. A BLASTx search (BLAST + v2.14.0) was performed on the *Ar. subalbatus* genome assembly against this curated database of viral proteins, employing an e-value threshold of 1e-6 [69]. The resulting hits were converted to BED format, sorted, and merged using BEDTools v2.31.0 [70]. The best hit from each cluster of overlapping hits was selected, and corresponding sequences were extracted from the genome assembly. To refine predictions and minimize false positives, a reverse BLASTx search of the extracted sequences was conducted against the NCBI nr protein database using DIAMOND v2.1.9 (e-value threshold: 1e-6) [71]. The resulting hits were utilized to recognize and remove false positives, including sequences with certain homology to eukaryotic proteins. Furthermore, viral integrations closer than 100 bp and derived from the same viral species were joined. Finally, each viral integration was assigned to a viral family based on its most similar virus using the VHost-Classifier tool [72].

## Results

### Genome assembly development, metrics, and features

**Fig. 1:**
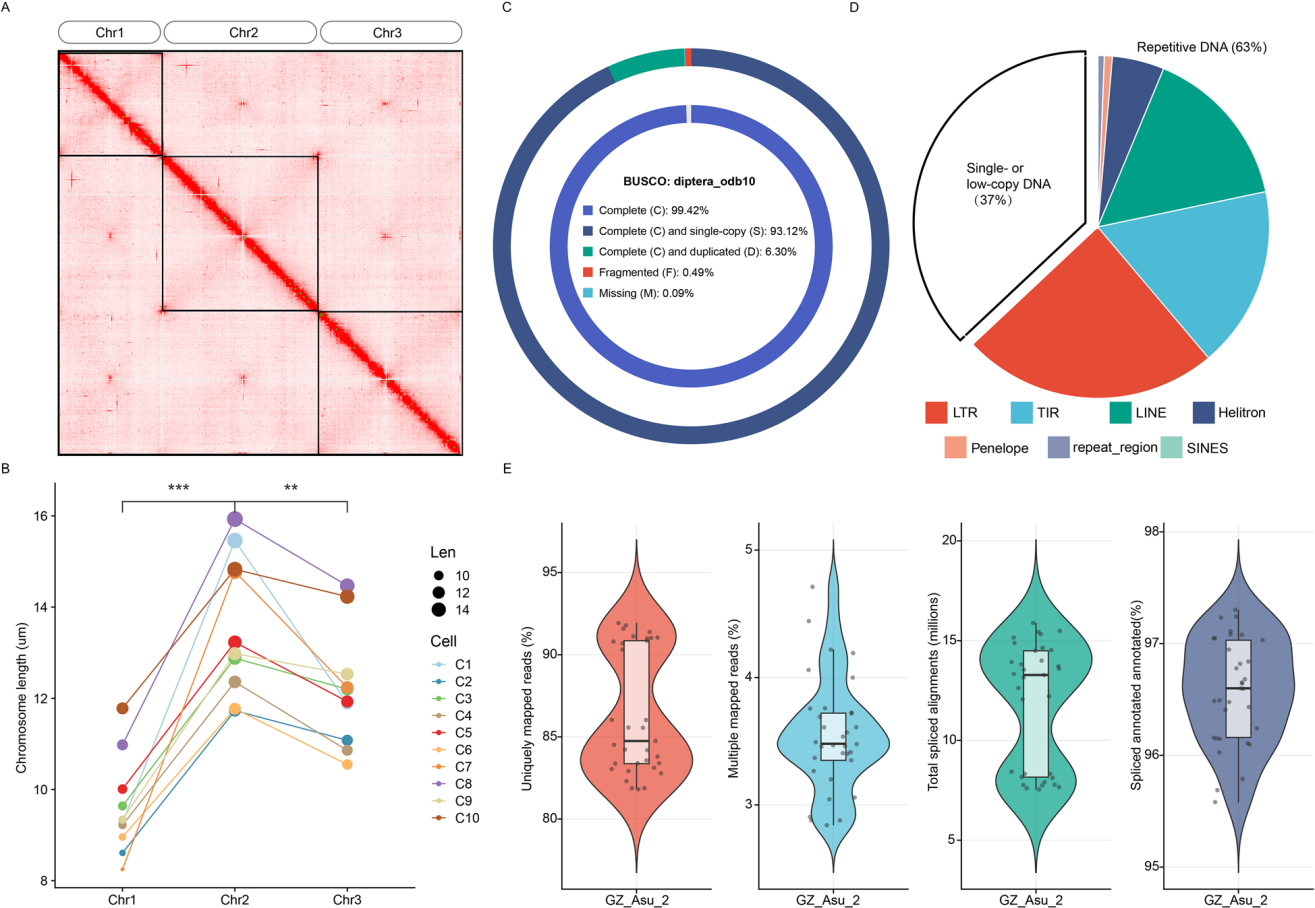
Genome assembly and characteristics of *Armigeres subalbatus*. **A** Hi-C contact map showing chromosome-level scaffolding of the genome assembly. **B** Chromosome length comparison across different cell types (C1–C10). *n* = 10. *** *P* < 0.001, ** *P* < 0.01. **C** BUSCO analysis with diptera_odb10 results showing genome completeness. **D** Pie chart illustrates the composition of repetitive elements in the genome. **E** Violin plots depicting various genomic features: Uniquely mapped reads (%), Multiple mapped reads (%), Total spliced reads (million), and Spliced alignments annotated (%). RNA-seq data were published at BioProject: PRJNA834573. LTR, long terminal repeat retrotransposon; TIR, terminal inverted repeat; SINES, short interspersed nuclear elements

The genome size of *Ar. subalbatus* is estimated to be between 1.21 and 1.38 Gbp, based on calculations using a previously reported C-value and GenomeScope analysis [73, 74]. To reduce heterozygosity, after six consecutive rounds of single sister-brother matings, we extracted high-molecular weight DNA from thirty sibling mosquitoes. We then generated approximately 160 Gb of Nanopore long reads with a mean read length of 23 kb and an N50 length of 28 kb (N50 length: half of the data comprises sequences of this length or longer). Additionally, we prepared a Hi-C proximity ligation library from thirty male pupae and collected 274 Gb of Illumina reads. We assembled the long-read ONT data with NextDenovo [31] and polished the resulting contigs with NextPolish [32] using the raw ONT data and NGS short reads. This initial assembly totaled 3.50 Gbp, to partition this initial assembly into primary and alternative contig sets, we analyzed contig alignments and depth of coverage with Purge_dups [33] to determine which contigs were likely to be redundant and should be designated as alternative alleles. Haplotig purging reduced the size of the primary assembly to 1.33 Gbp, which was then scaffolded via the Hi-C data using Juicer (v1.6) and 3D-DNA (v180922) pipelines [34, 35]. The Hi-C contact map demonstrates clear chromosomal structures, confirming the successful scaffolding of our assembly into three distinct chromosomes (Fig. 1A). The genome assembly of *Ar. subalbatus*, which we call GZ_Asu_2 (GenBank assembly: GCA_024139115.2), consists of 2078 scaffolds with an N50 length of 430.15 Mb (Additional file 2: Table S1), resulted in three major scaffolds with lengths of 465,049,604, 430,149,658, and 318,640,189 base pairs, respectively. These three scaffolds account for over 90% of the total genome length (Fig. 1A, Additional file 2: Table S1). Notably, the lengths of the two longest scaffolds are quite similar, which aligns with microscopic observations of chromosome lengths (Fig. 1B). To confirm the chromosomal identity and measure their relative lengths, we employed fluorescence in situ hybridization (FISH) using a 28S rDNA probe to specifically mark chromosome 3 [18, 75]. Subsequent measurements under optical microscopy revealed statistically significant differences in the lengths of the three chromosomes, confirming three distinct size classes that directly correspond to our genomic scaffolds (Fig. 1B). Quantitative analysis of 10 cells showed that chromosome 1 had a mean length of 9.61 ± 1.07 μm, chromosome 2 measured 13.53 ± 1.58 μm, and chromosome 3 was 12.27 ± 1.32 μm (Fig. 1B). Chromosome 2 was significantly longer than chromosome 3 (*P* < 0.01), and both chromosomes 2 and 3 were significantly longer than chromosome 1 (*P* < 0.001) (Fig. 1B). These cytological measurements validate our genomic assembly results, where the two longest scaffolds showed similar lengths (corresponding to Chr2 and Chr3), while the shortest scaffold corresponded to the distinctly smaller Chr1, providing independent confirmation of the chromosome-scaffold correspondence (Fig. 1B). The combination of bioinformatic analysis and cytogenetic observation provides strong evidence for the accuracy of our chromosome-level assembly, with the scaffold lengths closely corresponding to the physical lengths of the chromosomes observed microscopically. The genome assembly of *Ar. subalbatus* demonstrates high quality and completeness, comparable to other major mosquito species. BUSCO analysis using the diptera_odb10 lineage revealed 99.4% completeness (93.1% single-copy, 6.3% duplicated), with only 0.09% fragmented and 0.49% missing genes out of 3285 BUSCO groups (Fig. 1C, Additional file 2: Table S2).

Comprehensive analysis of the genome using the Extensive de novo TE Annotator (EDTA) revealed a substantial presence of transposable elements (TEs), which collectively amount to 63.0% of the total genomic sequence (Additional file 2: Table S3, Fig. 1D), a value comparable to that of the most recent assembly of the *Ae. aegypti* genome, AaegL5 [76]. The rate of alignments of DNA and RNA sequencing data from our published data (BioProject: PRJNA834573) [18] and the percentage of properly paired reads were analyzed and confirmed the quality and continuity of GZ_Asu_2. Quality assessment of the RNA-seq data demonstrated robust sequencing and alignment metrics across all samples (Additional file 2: Table S1, Fig. 1E). The distribution of uniquely mapped reads exhibited a bimodal pattern, with values ranging from 81.8% to 91.9%, indicating consistent but varying alignment efficiency across samples (Fig. 1E). Multiple mapped reads showed a relatively narrow distribution between 2.8% and 4.7%, suggesting well-controlled mapping specificity (Fig. 1E). The total number of spliced alignments varied considerably across samples, ranging from 7.551 M to 15.885 M, reflecting differences in splicing complexity among developmental stages (Fig. 1E). Notably, the percentage of annotated spliced alignments maintained a remarkably high and consistent level across all samples (95.6–97.3%), indicating that the vast majority of observed splicing events occurred at known splice junctions within the reference annotation (Fig. 1E). These quality metrics demonstrate the high technical quality and reliability of the RNA-seq dataset, providing a solid foundation for downstream analyses.

### Evolutionary origin and chromosomal context of the male-determining factor in *Armigeres subalbatus*

**Fig. 2:**
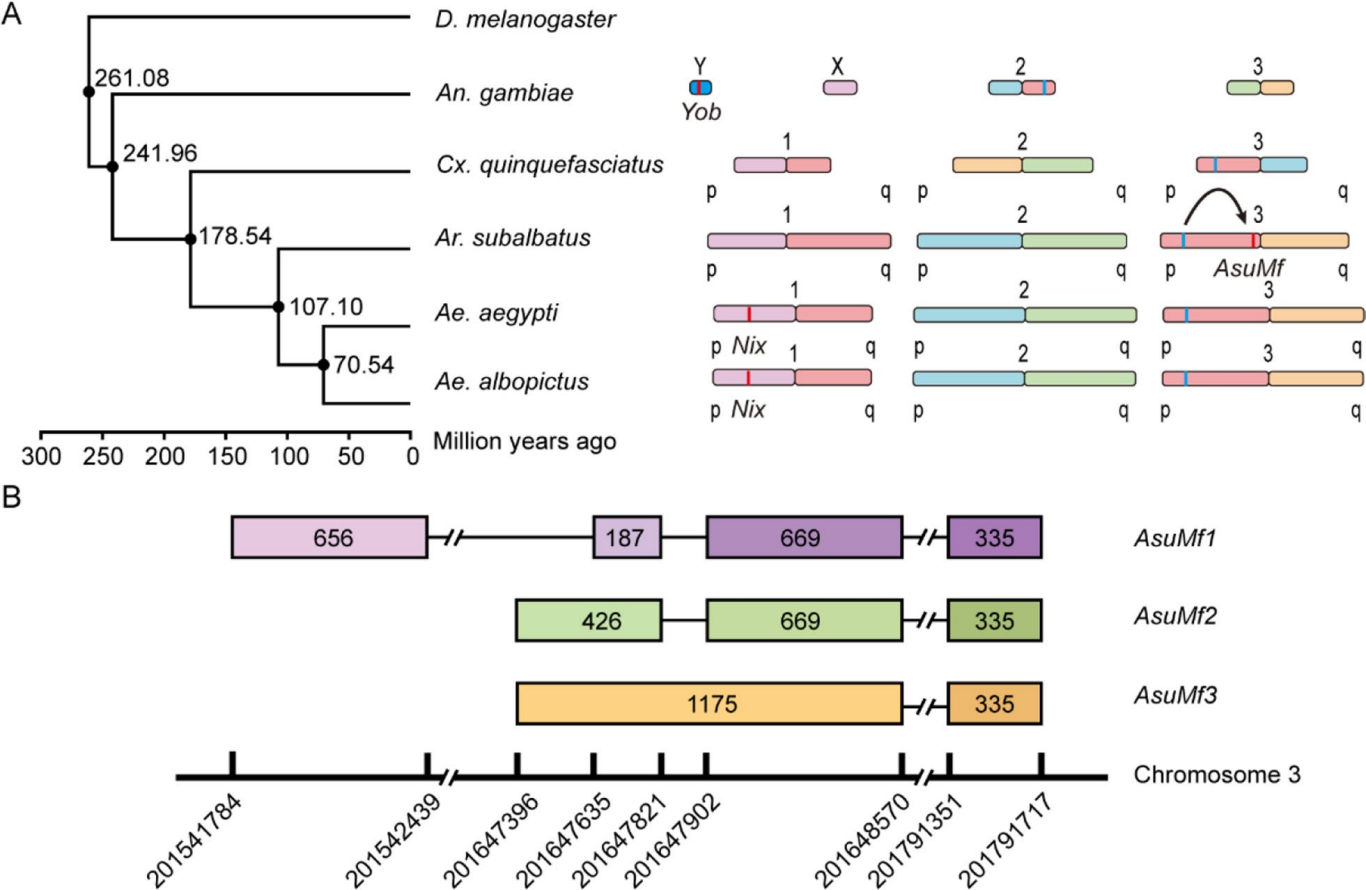
Chromosomal evolution of mosquito vectors and gene organization of the male-determining gene *AsuMf*. **A** Left: Time-calibrated maximum likelihood molecular phylogenetic tree of major mosquito vector species, including *Drosophila melanogaster* as an outgroup. Numbers at nodes indicate the estimated divergence time in million years ago, with 95% confidence intervals in parentheses. Right: Schematic representation of chromosome evolution across the studied species. Chromosomes are depicted as colored bars, with homologous regions across species shown in the same color. The sex-determining region (if known) is indicated for each species: Y chromosome (Y) in D. melanogaster, *Yob* in *An. gambiae*, and *Ni*x in *Ae. aegypti* and *Ae. albopictus*. In *Ar. subalbatus*, the newly identified male-determining factor *AsuMf* is shown on chromosome 3, along with its paralog *AsuHrp6*5. The variable position of the centromere is indicated by constrictions in the chromosomes.'p'and'q'denote the short and long arms of each chromosome, respectively. Arrow indicates the gene duplication event from ancestral *AsuHrp65* to *AsuMf*. **B** Schematic representation of the *AsuMf* gene locus on chromosome 3. The *AsuHrp65* paralog is shown upstream, with opposite orientation to *AsuMf*. Three isoforms of *AsuMf* (*AsuMf1*, *AsuMf2*, and *AsuMf3*) are depicted, illustrating their exon–intron structures. Exons are represented by colored boxes with their lengths in base pairs. Introns are shown as lines, with'//'indicating large introns not drawn to scale. Genomic coordinates are provided below

Phylogenetic analysis reveals that *Ar. subalbatus* diverged from the *Aedes* lineage approximately 107.10 million years ago (Fig. 2A). This divergence places *Ar. subalbatus* as a distinct clade, more closely related to *Aedes* than to *Culex* or *Anopheles*. Examination of chromosome structures across these species demonstrates significant genomic rearrangements throughout their evolution. Of particular interest is the presence of both *AsuHrp65* and *AsuMf* genes on the third chromosome of *Ar. subalbatus*. The positional relationship and sequence similarity, combined with synteny analysis between these genes strongly suggest that *AsuMf* originated from a duplication event of *AsuHrp65* (Fig. 2A) [18]. This duplication event may have occurred after the divergence of *Ar. subalbatus* from the *Aedes* lineage, indicating it is a relatively recent evolutionary innovation specific to the *Armigeres* genus (Fig. 2A). The subsequent neofunctionalization of *AsuMf* into a sex-determining factor represents a significant development in the evolution of sex determination mechanisms in this clade. This finding not only highlights the dynamic nature of sex determination systems in insects but also underscores the crucial role of gene duplication in driving evolutionary novelty. Further investigation into the molecular mechanisms and selective pressures that facilitated the functional divergence of *AsuMf* may provide valuable insights into the plasticity of sex determination pathways in vector mosquitoes.

The male-determining factor gene *AsuMf* in *Ar. subalbatus* is located on chromosome 3, spanning a significant portion of the genomic region from position 201,647,396 to 201,731,717, approximately 120 Mbp downstream from its paralog *AsuHrp65*, with the two genes arranged in opposite orientations. *AsuMf* exhibits a complex structure with three distinct isoforms (*AsuMf1*, *AsuMf2*, and *AsuMf3*), each displaying a unique exon-intron arrangement. All three isoforms share a common first exon of 335 bp. *AsuMf3*, the longest isoform, consists of two exons (335 bp and 1175 bp) separated by a 52,946 bp intron. *AsuMf2* comprises three exons (335 bp, 669 bp, and 426 bp), with introns of 52,946 bp and 80 bp. *AsuMf1*, the most structurally complex isoform, is composed of four exons (335 bp, 669 bp, 187 bp, and 656 bp) separated by introns of 52,946 bp, 80 bp, and 28,783 bp, respectively. This complex gene structure with multiple isoforms and varying intron lengths implies sophisticated regulation of the male-determining factor in *Ar. subalbatus*, potentially allowing for fine-tuned control of sex determination processes (Fig. 2B).

### Structure of the male determining locus

**Fig. 3:**
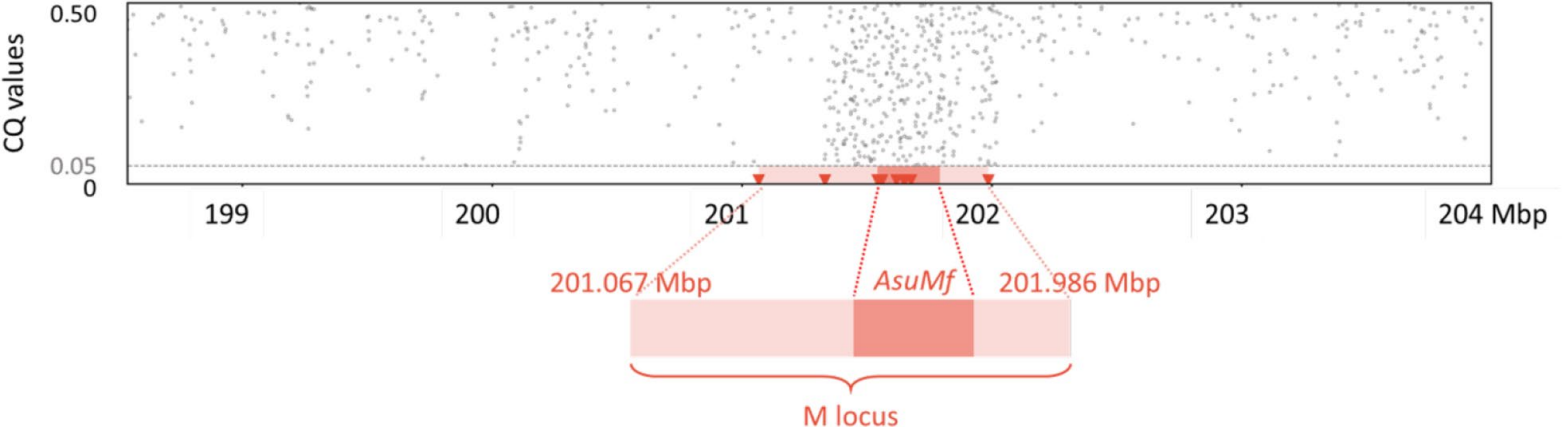
Chromosome quotient (CQ) analysis of genomic DNA from male and female libraries aligned to *A. subalbatus* genome. Each dot represents the CQ value of a repeat-masked 500-bp window with > 20 reads aligned from male libraries. The 500-bp windows with CQ ≤ 0.05, *i.e.* male-biased sequences, were shown as red triangles, and those with 0.05 < CQ ≤ 0.50 were colored grey, and the rest with CQ > 0.50 were ignored. All the male-biased sequences distribute in the region from 201.067 to 201.986 Mbp, including the male-determining gene *AsuMf*, which indicates this region is the M locus

Sex determination in *Ar. subalbatus* is governed by a dominant male-determining factor (M factor) at a male-determining locus (M locus) on chromosome 3 [77], similar to *Aedes* [78] and *Culex* [79]. *Ar. subalbatus* chromosome 3 is homomorphic between sexes except for the M/m karyotype, meaning that males are M/m and females are m/m. Our previous study showed that the male-determination gene, *AsuMf*, locates in the centromere on chromosome 3 [18]. We identified a region that contains *AsuMf* from 201.067 Mbp to 201.986 Mbp on chromosome 3 using the chromosome quotient (CQ) analysis [80], is the designation for the M locus (Fig. 3). The size of the *A. subalbatus* M locus is estimated at approximately 900 kb. Since *AsuMf* is derived from duplication and subsequent neofunctionalization of a conserved gene family, the M locus encompassing the *AsuMf* gene is a region of reduced recombination, and this peculiarity is quite similar to *Ae. aegypti* M locus [76]. Except for *AsuMf*, no other coding gene was identified in the M locus. More than 44.8% of the M locus is repetitive: long terminal repeat retrotransposons comprise 23% of the M locus compared to 3.9% genome-wide. Of these transposon elements in the M locus, the “BEL/Pao” elements account are the most abundant, accounting for approximately 11.3% of the M locus. The differentially accumulation of the repetitive sequences between the M locus and other regions occurred as a result of the sexually antagonistic mutations, matched with male-limited transmission [81]. The content and structure of M locus in mosquito genera is totally different. First, only one coding gene, *i.e. AsuMf*, was found in *Ar. subalbatus* M locus [18], while two coding genes, *i.e. Nix* [14] and *myo-sex* [82], were characterized in *Ae. aegypti* M locus. Second, the composition of their transposon element families is completely different. It indicates that M locus in divergent mosquito species evolved independently and rapidly [11].

### Chemosensation and xenobiotic detoxification

**Fig. 4:**
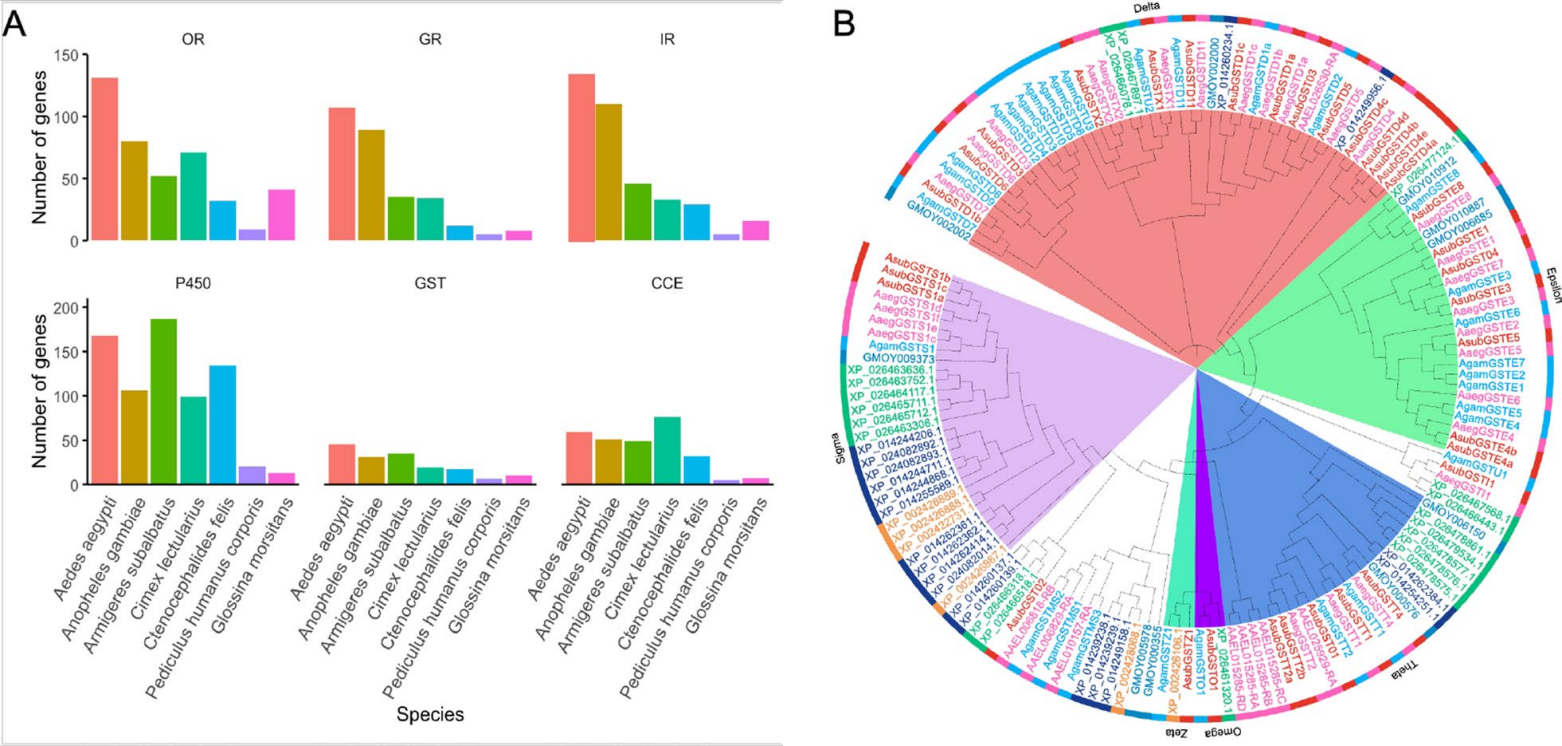
Comparison of chemosensory and detoxification enzymes genes among winged and wingless haematophagous insects. **A** Gene numbers of major chemosensory receptors and detoxification enzymes in winged mosquitoes and other wingless haematophagous insects. The InterPro was utilized to annotate the candidate genes of chemosensation and detoxification enzymes. Amino acid sequences were aligned using MAFFT with poorly aligned regions removed through trimAl using the “automated” option [46]. **B** Phylogenetic relationship of GST genes across *Ae. aegypti*, *An. gambae*, *Ar. subalbatus*, *C. lectularius*, *C. felis*, *P. humanus corporis* and *G. morsitans*. Phylogenetic trees were constructed by FastTree [85]. The Interactive Tree of Life (iTOL) was adopted for visualisation and annotation of the trees [61]

Chemosensensory (olfactory and gustatory) process in the insect peripheral nervous system depends on three families of receptors, including odorant, gustatory and ionotropic receptors. Odorant receptors (ORs) play vital roles in host searching, habitat navigating and oviposition site location through the detection of semiochemicals. The major functions of gustatory receptors (GRs) are to mediate gustation—most importantly to detect sweet and bitter tastants—as well as to sense carbon dioxide, which is also critical for the host seeking process of haematophagous mosquitoes. Ionotropic receptors (IRs) evolved from ionotropic glutamate receptors in ancestral animals, and display versatile roles in multiple physiological activities, including but not limited to olfaction and gustation. In *Ar. subalbatus*, we identified 52 OR, 35 GR, and 46 IR genes (Fig. 4A). This repertoire of chemosensory genes is comparable to the malaria mosquito *Anophele gambiae* and slightly reduced when compared to *Aedes aegypti.* However the chemosensory receptors of *Ar. subalbatus* mosquito are substantially enriched when compared to those wingless haematophygous insects, such as the common bed bug (*Cimex lectularius*), cat flea (*Ctenocephalides felis*), or body louse (*Pediculus humanus corporis)*, which is reasonable when considering the much broader chemical space of the winged *Armigeres* mosquito that have a wide host spectrum than those narrowly tuned blood-feeding insects which are usually host-specialized and haematophagous obligated. Moreover, we found three CO_2_ receptors (Gr1, 2, 3, one to one ortholog of *Ae. aegypti* Gr1, 2, and 3) with Gr1 extensively expressed across different life stages/tissues of *Armigeres* that renders us to speculate that Gr1 may have additional functions beyond CO_2_ detection (Additional file 1: Figure S2). While no sugar receptors have been found in several obligate blood-feeding insects (e.g. bed bugs, tsetse fly and lice), *Armirgeres* mosquitoes have a complete repertoire of sugar receptors just as the other mosquitoes, which is in line with their nectar/honeydew-feeding feature. It is worth to mention that *Armigeres* genome misses the Gr6 lineage of sugar receptors and have a dramatically reduced number of bitter receptors that may have a significant impact on its feeding biology, particularly detecting sensing some toxic or unpleasant compounds in the foods (Additional file 1: Figure S3). We also find that IR genes are dramatically reduced with only 45 IRs identified in *Amigeres* mosquitoes wherease *Ae, aegypti* or *An. gambiae* own > 100 IRs in their genome. The shrinkage of *Amigeres* IRs may limit their coding capacity to the amines or acids released from human or animal hosts.

Detoxification enzymes play critical roles in the xenobiotic metabolism of insects. There are three major groups of enzymes associated with xenobiotics degradation: cytochrome P450s, carboxylesterase and glutathione s-transferase (GST). The largest detoxification enzyme group in *Ar. subalbatus* is the cytochrome P450s, for which a total of 187 genes were identified. This represents a significant expansion of P450s relative to other haematophagous insect species, in which *Ae. aegypti* and *An. gambiae* have 128 and 105 P450 genes respectively. Most of the predicted cytochrome P450 genes (172 genes) have full length while 15 genes are annotated as pseudogene. The most predominant P450 families in *Ar. subalbatus* are CYP4 and CYP6, which contain 57 and 52 genes, respectively, and represent > 58% of all cytochrome P450s in the *Ar. subalbatus* genome (Additional file 1: Figure S4). The expansion of cytochrome P450 genes in *Ar. subalbatus* mainly happens within clan 3 (including CYP6 and 9) and 4 (58 and 88 genes, respectively) which are considered to be related to the xenobiotic metabolism and pesticide resistance as previous studies demonstrated [83, 84]. Intriguingly, the gravid female *Ar. subalbatus* are often found to preferentially oviposit in foul water, such as cesspool and manure pit that are usually heavily contaminated by microbial metabolic substances and/or low pH acids. It is possible that expansion of P450 genes enables larval mosquitoes feeding in such a “dirty” environment to equip a much stronger detoxification capacity to tolerate the chemical/acid stresses so as to fulfill their growth and development. Collectively, the complement of cytochrome P450 genes identified in the *Ar. subalbatus* mosquitoes consist of those anticipated to be present, along with a predominance and expansion of cytochrome P450 genes from the CYP3 and CYP4 families which may relate to their survival in the harsh living environment.

A total of 35 GST genes are predicted from the *Ar. subalbatus* genome. This is similar to the number present in *An. gambiae* which has 31 GST genes; and fewer than that in *Ae. aegypti*, which has 45 GST genes. A phylogenetic analysis of the GSTs for *Ar. subalbatus*, *Ae. aegypti*, and *An. gambiae* showed that the *Ar. subalbatus* GST genes were distributed among the different classes (epsilon, omega, theta, sigma, and zeta) of cytosolic GSTs which are also present in other mosquito species (Fig. 4B). An additional 4 microsomal GST genes were predicted from the *Ar. subalbatus* genome, which is similar to the number of microsomal GST genes present in An. gambiae (three genes with a total of four isoforms). Moreover, a total of 49 genes were predicted to have esterase activities, including acetylcholinesterase, thioesterase, carboxylesterase, neuropathy target esterase and palmitoyl-protein thioesterase.

### Diapause-related genes

Diapause in insects is a hormonally programmed state of dormancy, distinguished by its anticipation of adverse environmental conditions and its persistence even upon the resurgence of favorable ones [25]. In mosquitoes, diapause can manifest at various life stages, encompassing the embryonic, larval, or adult phages [25]. Similar to the genetic insect model, *Drosophila melanogaster*, the diapause phenomenon in *An. gambiae* and *Cx. pipiens* is identified at the adult stage, whereas in *Ae. albopictus*, it occurs at the egg stage. Conversely, *Ae. aegypti* exhibits non-diapause behaviour [25, 86]. In 1992, in the temperate confines of Mengshan area in Shandong Province, China, *Ar. subalbatus* was observed capable of overwintering through larval diapause [87]. These observations underscore the remarkable adaptability exhibited by these insects, enabling them to effectively navigate seasonal or environmental fluctuations through complicated regulatory mechanisms associated with diverse diapause-related pathways. The intricate interplay of these pathways underscores the insects'capacity to dynamically adjust their life cycles and achieve territorial expansion in response to changing environmental cues, demonstrating a high degree of physiological flexibility and resilience.

**Fig. 5:**
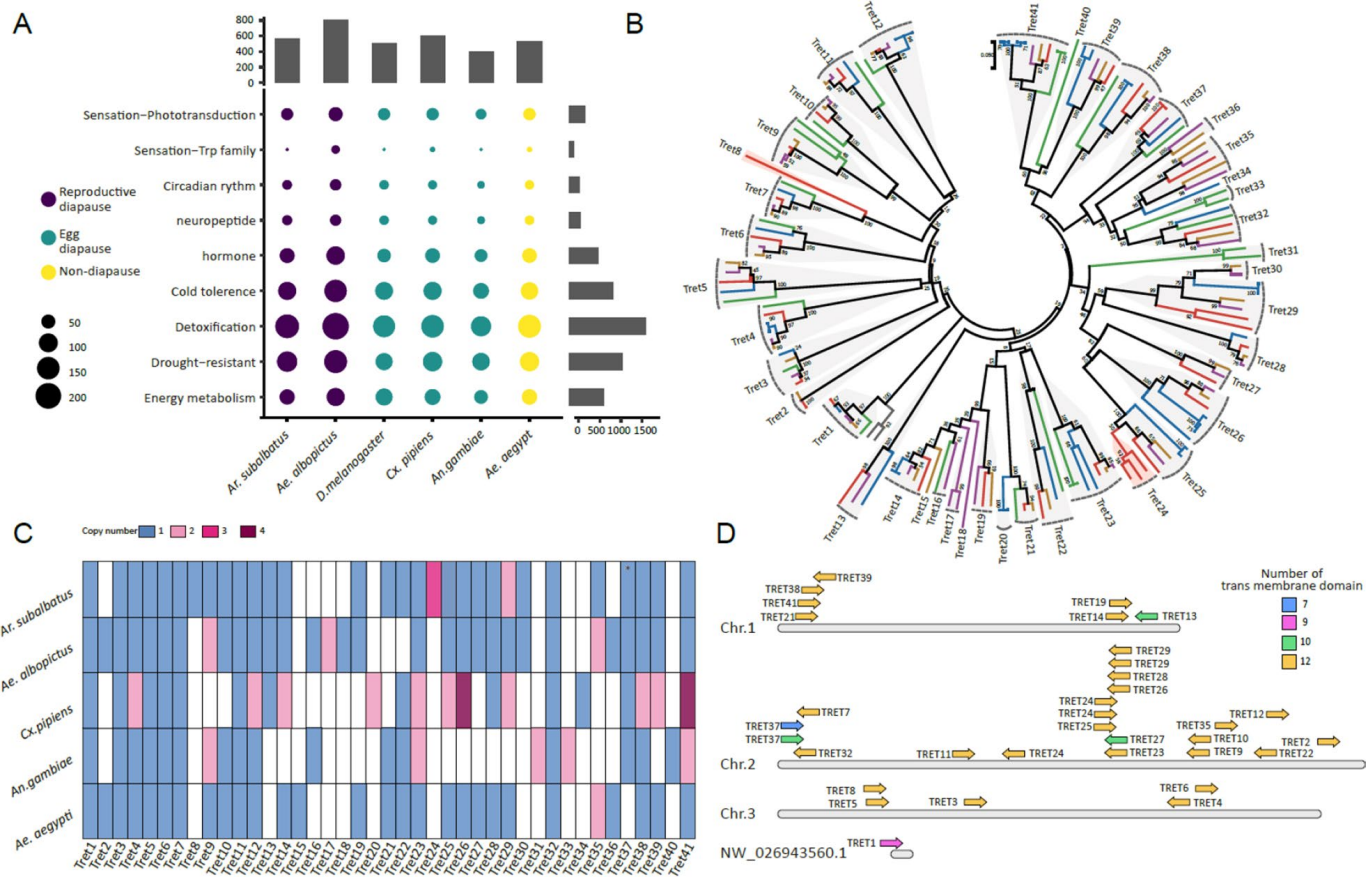
Analysis of putative diapause-related genes in the genome of *Ar. subalbatus*. **A** A comparison of the number of diapause-related genes in each group within *Ar. subalbatus* was conducted with those observed in *Ae. albopictus* (exhibiting diapause at the egg stage [93], *D. melanogaster and Cx. pipiens* (exhibiting diapause at the adult stage [94, 95], *An. gambiae* (exhibiting migration at wet season in long distance [96], as well as *Ae. aegypti* (a non-diapause species). **B** A phylogenetic analysis of Trehalose TRETs was conducted, incorporating the amino acid sequences of TRETs from the aforementioned six species. Through this phylogenetic analysis, TRETs were categorized into 40 variants. The branches of the phylogenetic tree are colored differently to represent the different species. **C** The distribution of TRET variants across the five mosquito species was assessed. **D** The different TRET variants with different transmembrane region numbers located in *Ar. subalbatus* genome

Based on the aforementioned nine gene sets of putative diapause-related genes in insects, we identified a comprehensive total of 566 genes related to diapause that have been annotated within the genome of *Ar. subalbatus* (Fig. 5A, Additional file 2: Table S4). When comparing the aggregate number of putative diapause-related genes across all nine groups and the respective counts within each group in *Ar. subalbatus* with those in the other five species, although no statistically significant differences were discernible, suggesting a genomic stability in terms of gene counts underlying the regulation of insect diapause or quiescence behaviors, we nevertheless noted a prominent representation of four groups of diapause-related genes in the *Ar. subalbatus* genome, similar to that observed in *Ae. albopictus* and *Cx. pipiens.* These groups include Detoxification, Cold Tolerance, Drought-Resistance, and Energy Metabolism (Fig. 5A).

The Trehalose Transporter (TRET) family plays a pivotal role in insect diapause metabolism. Upon conducting a thorough phylogenetic analysis, we have determined that the TRETs within six aforementioned insect species can be systematically classified into a total of 41 variants (Fig. 5B). The specific distribution of TRETs across the five mosquito species was assessed as follows: 34 variants in *Ar. subalbatus*, 34 in *Ae. albopictus*, 38 in *Cx. pipiens*, 26 in *An. gambiae*, and 29 in *Ae. aegypti* (Fig. 5C). Notably, among these diapause-exhibiting species, *Ar. subalbatus* uniquely harbored TRET29, TRET24, and TRET8 with copy numbers of 2, 3, and 1, respectively. Similarily, *Ae. albopictus* was found to uniquely possess TRET18, (with 1 copy) and TRET17 (with 2 copies). Meanwhile, *Cx. pipiens* and *An. gambiae* uniquely harbored TRET20 (with 2 copy), TRET34 (with 1 copy) and TRET31, TRET33 (both with 2 copies), respectively. Additionally, TRET37 with two isoforms was consistently present in all four diapause-exhibiting mosquito species but absent in the non-diapause species, *Ae. aegypti*, indicating a diapause-related gene expression pattern (Fig. 5C). Furthermore, an examination of mosquito species has revealed the presence of twelve transmembrane domains in TRETs. Specifically, within the species *Ar. subalbatus*, TRET variants display four distinct types, characterized by 7, 9, 10, and 12 transmembrane domains respectively, with the majority possessing 12 transmembrane domains. Interestingly, TRET8, a variant uniquely harbored by *Ar. subalbatus,* lacks the transmembrane domain (Additional file 1: Figure S5). Moreover, the majority of TRET variants in *Ar. subalbatus* are situated on chromosome 2, with TRET29 and TRET24 possessing 2 and 3 copies respectively, and TRET37 exhibiting 2 isoforms. Meanwhile, TRET8 is localized on chromosome 3 (Fig. 5D)*.*

During the diapause induction and preparatory phase, insects undergo significant lipid accumulation to sustain the metabolic demands of diapause and to guarantee subsequent normal developmental processes. Lipase3 was found mediating Drosophila metabolic responses to starvation and longevity [88, 89]. Through a comprehensive phylogenetic analysis, we have delineated the existence of 13 distinct variants of Lipase3 across the aforementioned six insect species (Additional file 1: Figure S6A). Interestingly, analogous to TRET37, which displays a diapause-associated gene expression pattern, Lipase3-4 is uniformly present in all four diapausing mosquito species but notably absent in the non-diapausing *Ae. aegypti*. Conversely, Lipase3-7 is consistently absent in these four diapausing species but present in *Ae. aegypti* (Additional file 1: Figure S6B). These data indicate that distinct Lipase3 variants may play important roles in regulating mosquito diapause. Furthermore, the majority of Lipase3 variants, including Lipase3-4 in *Ar. subalbatus*, were found to be located on chromosome 1 (Additional file 1: Figure S6C).

Insights into insect circadian rhythms originated from studies on *D. melanogaster*, where the transcription repressors Period (PER) and Timeless (TIM) inhibit the activators Clock (CLK) and Cycle (CYC), constituting the primary TTFL. In contrast, mammals employ a distinct mechanism involving CLK-BMAL1(brain and muscle arnt-like 1) suppression through PER and Cryptochrome (CRY), forming the core of their circadian system. Notably, the monarch butterfly (*Danaus plexippus*), known for its migratory behavior, utilizes a unique TTFL where TIM, PER, and CRY2 form trimer to inhibit CLK-CYC [90]. Meanwhile, CRY1 of *D. plexippus*, encoded by the *cry*1 gene, functions as a circadian photoreceptor facilitating TIM degradation upon light exposure [90].

To investigate the circadian feedback loop in *Ar. subalbatus*, we annotated key circadian genes, regulators, and signal output genes (Additional file 2: Table S5), and conducted a phylogenetic analysis of the cry gene (Additional file 1: Figure S6). Our findings revealed the conservation of circadian genes across mosquito species. Phylogenetic analysis of the *cry* gene indicated that *Ar. subalbatus*, like *D. plexippus* and *Bombyx mori*, possesses both *cry*1 and *cry*2 (Additional file 1: Figure S7). CRY1 is crucial in seasonal rhythm regulation, as evidenced by shift the diapause phenotype in *B. mori* following *cry*1 knockout [91]. Meanwhile, knockout of *B. mori cry2* lead to a reduction of diapause incidence [92]. Among the circadian clock genes in *Ar. subalbatus*, *Clk*, *per*, *sgg*, and *vrille* showed the closest phylogenetic relationship to those in *Ae. aegypti*, whereas *tim* and *doubletime* (*dbt*) were most related to those in *Ae. albopictus*. Notably, each of these genes is represented by a single copy in the genome of *Ar. subalbatus* but exhibits multiple transcript variants, including four for *tim*, two for *per*, five for *cyc*, and four for *dbt* (Additional file 2: Table S5).

### Innate immune system

**Fig. 6:**
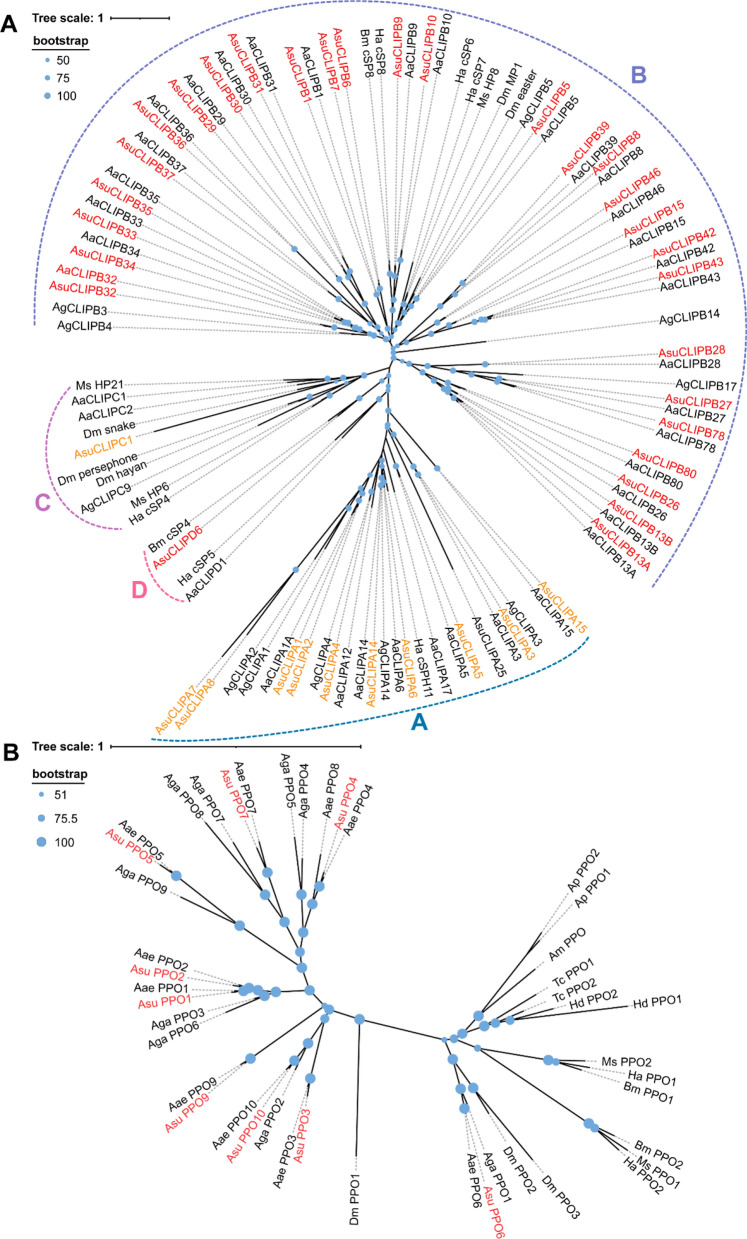
The CLIPs and PPOs of *Ar. subalbatus*. **A** Phylogenetic analysis of CLIPs. The amino acid sequences of 6 *Helicoverpa armigera* (Ha), 5 *D. melanogastor* (Dm), 2 *B. mori* (Bm), 3 M*. sexta* (Ms), 11 *An. gambiae* (Aga), 38 *Ae. aegypti* (Aae) and 41 *Ar. subalbatus* (Asu) SP domains were compared. The CLIPs are divided into four subgroups. Scale bar, 1 substitution per site. Blue dots at nodes indicate bootstrap values greater than 50% from 1,000 resamples. All clip domain-SPs of *Ar. subalbatus* are shown in red and clip domain-SPHs showed in orange. **B** Phylogenetic analysis of PPOs. The amino acid sequences of 2 *Acyrthosiphon pisum* (Ap), 2 *H. armigera* (Ha), 2 T*. castaneum* (Tc), 3 *D. melanogastor* (Dm), 2 *B. mori* (Bm), 1 *A. mellifera* (Am), 2 *Holotrichia diomphalia* (Hd), 9 *An. gambiae* (Aga), 10 *Ae. aegypti* (Aae) and 10 *Ar. subalbatus* (Asu) PPOs were compared. Scale bar, 1 substitution per site. Blue dots at nodes indicate bootstrap values greater than 51% from 1,000 resamples. All PPOs of *Ar. subalbatus* are shown in red

Mosquitoes utilize distinct immune response pathways for parasites, primarily involving melanization [97, 98], and for viruses [99], relying on RNA interference (RNAi) [99] and the JAK/STAT [100] signaling pathway. Comparatively, 334 innate immune genes were annotated in *Ar. subalbatus*, a number higher than those in other model species or vector insects (Additional file 2: Table S6). For instance, immune genes number of *D. melanogaster*, *An. gambiae*, *Ae. aegypti*, *B. mori*, *A. mellifera*, and *T. castaneum* are 267, 283, 349, 167, 114, and 234, respectively (Additional file 2: Table S7). *Ar. subalbatus* showed expansions in several immune gene families crucial for pathogen recognition such as PGRP, *β*GRP/GNBP, Galectin, C-type lectin, FREP, TEP, and SCR (Additional file 1: Figure S8), as well as genes involved in IMD and Toll signaling pathways (Additional file 1: Figure S9-S10). These expansions highlighted *Ar. subalbatus*'s robust immune capabilities as a vector capable of pathogen transmission. However, compared to the closely related vector *Ae. aegypti*, *Ar. subalbatus* had undergone contractions in certain gene families like clip-domain serine proteases (CLIPs) and their specific inhibitors, serpins (Fig. 6 and Additional file 1: Figure S11), affecting the modulation of melanization which is a crucial component of their innate immune system [53, 101] in invertebrates and regulated by a series of CLIPs [102–104] and serpins [105, 106]. The melanization process begins with the proteolytic activation of inactive precursor prophenoloxidase (PPO) by, resulting in the formation of phenoloxidase (PO), which catalyzes the transformation of monophenols into o-diphenols and subsequently oxidizes these o-diphenols into o-quinones [97, 107], and a relative deficiency in melanization response potentially reduced its resistance to parasites, as also observed in *An. gambiae*, a host of *Plasmodium* parasites [108, 109]. Nevertheless, *Ar. subalbatus* compensated with a stronger sensitivity in pathogen recognition and a more complex RNAi pathway (Additional file 2: Table S7), resulting in fewer viruses both in terms of types and quantities within its body. These insights indicated a coordinated pattern of gene family expansions and contractions in innate immune system of *Ar. subalbatus*'s, aligning with its dual role as both a virus vector and a host for parasites.

### Identification and characterization of endogenous viral elements

The *Ar. subalbatus* genome was analyzed against the NCBI viral protein Refseq databases, and the E-value threshold was set to 1 × 10^–6^, yielding 13,929 candidate fragments. These fragments were subsequently compared against NCBI’s non-redundant (NR) database to exclude sequences with high identity to host proteins and false positives, resulting in 1673 confirmed EVE candidate fragments.

**Fig. 7:**
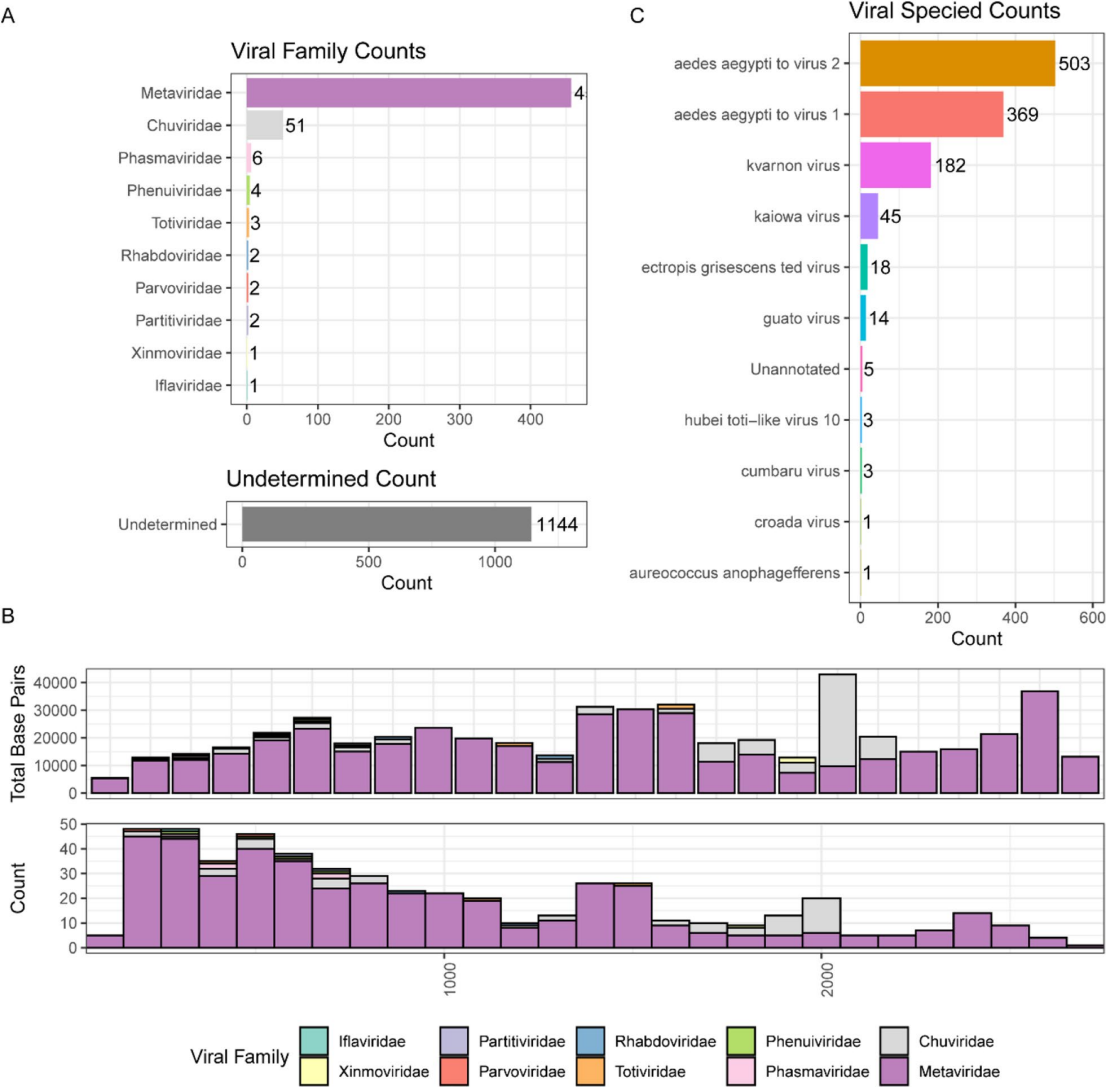
Classification and analysis of endogenous viral elements (EVEs) in *Ar. subalbatus*. **A** Of the identified EVEs, 530 were classified into 10 distinct families, with their frequencies displayed on the x-axis (upper panel). An additional 1144 EVEs remained unclassified and are analyzed separately (lower panel). **B** The size distribution of EVEs is presented as a histogram showing both the total base pairs per size category (upper panel) and the frequency distribution of EVE lengths in the *Ar. subalbatus* genome (lower panel). **C** The unclassified EVEs (n = 1144) were further analyzed for their characteristics, with their distribution illustrated on the x-axis (upper panel)

Among these, 530 EVEs were classified into 10 viral families (Fig. 7A): *Metaviridae*, *Chuviridae*, *Phasmaviridae*, *Phenuiviridae*, *Totiviridae, Rhabdoviridae*, *Parvoviridae*, *Partitiviridae*, *Xinmoviridae*, and *Iflaviridae.* Of these families, only *Parvoviridae* represents DNA viruses (linear, single-stranded), while the remainder are RNA viruses. The identified EVEs spanned 13,090,278 bp, ranging from 26 to 6503 bp in length, with a median length of 755 bp (Fig. 7B). Detailed taxonomic distribution and characteristics of these 530 EVEs are presented in Additional file 2: Table S8.

*Metaviridae* emerged as the most prevalent viral family, comprising 457 EVE integrations. This family of retrotransposons and reverse-transcribing viruses, belonging to the order *Ortervirales*, contains long terminal repeats. The genera *Errantivirus* and *Metavirus* are distinguished by the presence or absence of the env gene, respectively, with examples including *Saccharomyces cerevisiae* Ty3 virus and its Gypsy-like relatives in *Drosophilids* [110]. Notably, many invertebrate *Metaviridae* contain an env-like third ORF, resembling vertebrate endogenous retroviruses, with DmeGypV (*Drosophila* Gypsy endogenous retrovirus) serving as the primary model [111].

*Chuviridae* represented the second most abundant family with 51 EVEs. Current knowledge of this family is limited to its arthropod host range, variable genomic structure (unsegmented, bisegmented, and circular), and EVE presence across various arthropod orders [112, 113]. *Chuvirus*-derived EVEs are widely distributed throughout the *Culicidae* family, suggesting integration predating the divergence of *Culicinae* and *Anophelinae* subfamilies. These EVEs potentially serve dual functions: enabling *chuvirus* glycoprotein endogenization into Pao retrotransposons, and facilitating antiviral responses against *chuviruses* and *Anakin retroviruses* [113].

The remaining 1144 unclassified EVEs demonstrated notable alignments with previously identified viruses. The most abundant aligned with Aedes aegypti To virus 2 (AAToV2; 503 EVEs) and Aedes aegypti To virus 1 (AAToV1; 369 EVEs), first identified in Brazilian *Ae. aegypti* populations and classified as *Metaviridae* family members [114]. The third most abundant aligned with Kvarnon virus, observed in *Aedes communis*, a prevalent northern European species frequently parasitized by water mites. This virus-host relationship suggests potential viral transfer between mosquitoes and mites during their symbiotic interactions [115].

## Discussion

The high-quality chromosome-level genome assembly of *Ar. subalbatus* presented in this study provides new insights into the genomic architecture and molecular evolution of this emerging disease vector. Our assembly demonstrates high completeness with 99.4% BUSCO score, which is comparable to *Aedes aegypti* (99.7%) and *Anopheles gambiae* (99.9%), and superior to *Aedes albopictus* (98.1%) and *Culex quinquefasciatus* (95.9%) (Additional file 2: Table S2). These quality metrics, combined with the chromosome-level scaffolding, enable comprehensive comparative genomic analyses across major mosquito vector species.

The substantial TE content (63.0% of the genome) observed in *Ar. subalbatus* provides insights into the evolutionary dynamics of mosquito genomes. This high TE content may have significant implications for genome size evolution, gene regulation, and genomic plasticity, potentially contributing to the organism's adaptability and evolutionary potential. The observed TE composition and distribution pattern provide valuable insights into the evolutionary history and genomic architecture of *Ar. subalbatus*, highlighting the dynamic nature of mosquito genomes and their capacity for structural reorganization through transposable element activity.

Our chromosome-level genome assembly enabled detailed characterization of *AsuMf* gene structure, providing new insights into this previously identified male-determining factor that evolved from *AsuHrp65* duplication [18]. This represents a distinct evolutionary trajectory from male-determining factors like *Nix* in *Aedes* and *Yob* in *An. gambiae* [12, 14, 16, 18]. The presence of multiple *AsuMf* isoforms suggests complex sex determination regulation, though their functional roles require further validation. Our analysis revealed substantial expansion of detoxification-related genes, particularly cytochrome P450s (187 genes compared to 128 in *Ae. aegypti* and 105 in *An. gambiae*) [76]. The expansion of CYP4 and CYP6 families likely reflects adaptation to polluted breeding sites [116], while reduced bitter receptors and IR genes suggest potential host-seeking behavior specialization.

The identification of 566 putative diapause-related genes, including an expanded Tret family, provides molecular insights into the species'unique larval diapause strategy, distinct from the egg diapause in *Aedes* or adult diapause in *Culex* mosquito [25, 26]. The immune system architecture shows notable differences from other mosquito vectors, with expanded pathogen recognition genes but contracted melanization pathway components [117]. The extensive collection of endogenous viral elements (1673 EVEs), indicating a complex and enduring history of virus-host interactions. These integrated viral sequences are not merely genomic remnants; they actively contribute to the mosquito's antiviral immunity, primarily through piRNA production. This mechanism, where EVEs participate in host defense, offers critical insights into *Ar. subalbatus*'s vector competence and informs the development of novel vector control strategies [24, 118].

While our high-quality genome assembly advances understanding of mosquito evolution and adaptation, some limitations exist. Certain repetitive regions remain challenging to resolve completely, and many identified genetic features require experimental validation. Nevertheless, our findings provide potential targets for vector control strategies [119], particularly relevant given *Ar. subalbatus*'s expanding geographical range and vector capacity. The unique adaptations identified demonstrate the importance of studying diverse vector species and establish a foundation for developing targeted control strategies against this emerging disease vector.

## Conclusions

In this study, we successfully assembled a high-quality, chromosome-level genome for the vector mosquito *Ar. subalbatus*, comprising three chromosomes with a total size of 1.33 Gb, an N50 of 430.15 Mb, and 99.4% BUSCO completeness. Comparative genomic analyses elucidated the gene structure of the male-determining factor (*AsuMf)* and its evolutionary relationship with other mosquito vectors. Notably, we identified significant expansions in detoxification-related gene families, particularly cytochrome P450s, which likely facilitate the adaptation of *Ar. subalbatus* to contaminated breeding sites. Furthermore, the characterization of 566 putative diapause-related genes, 334 innate immune genes, and 1,673 endogenous viral elements provides critical insights into its geographical expansion capabilities and complex virus-host interactions throughout its evolution. Collectively, these findings significantly advance our understanding of the molecular basis underlying vector competence and environmental adaptation in *Ar. subalbatus*, thereby establishing a crucial genomic foundation for the development of novel and targeted vector control strategies against this emerging disease vector.

## Supplementary Information

Additional file 1.

Additional file 2.

## Data Availability

All the data that support the genome assembly described in this study have been deposited in the NCBI repository and can be accessed with the BioProject accession ID PRJNA834573.

## Abbreviations

BUSCO: Benchmarking Universal Single-Copy Orthologs
NGS: Next generation sequencing
ONT: Nanopore sequencing
ESTs: Expressed sequence tags
SRA: Sequence read archive
LTR: Long terminal repeat retrotransposon
TIR: Terminal inverted repeat
SINES: Short interspersed nuclear elements
EVEs: Endogenous viral elements
M locus: Male-determining locus
CQ: Chromosome quotient
GRs: Gustatory receptors
ORs: Odorant receptors
GST: Glutathione s-transferase
PER: Period
TIM: Timeless
CLK: Clock
CYC: Cycle
CLIPs: Clip-domain serine proteases
PPO: Precursor prophenoloxidase
PO: Phenoloxidase
