# Supplementary material for: Insights on the sex determination, vector capacity and ecological biology from a chromosomal level genome of vector mosquito, Armigeres subulbatus
Source: Infect Dis Poverty. 2025 Aug 8;14:84. doi: 10.1186/s40249-025-01353-1 (PMC12333114; doi:10.1186/s40249-025-01353-1)
Supplement: Supplementary file 1 — Additional file 1. [file 40249_2025_1353_MOESM1_ESM.docx]

**Supplementary information**

**Additional file 1 includes supplementary Figure S1 to S10**


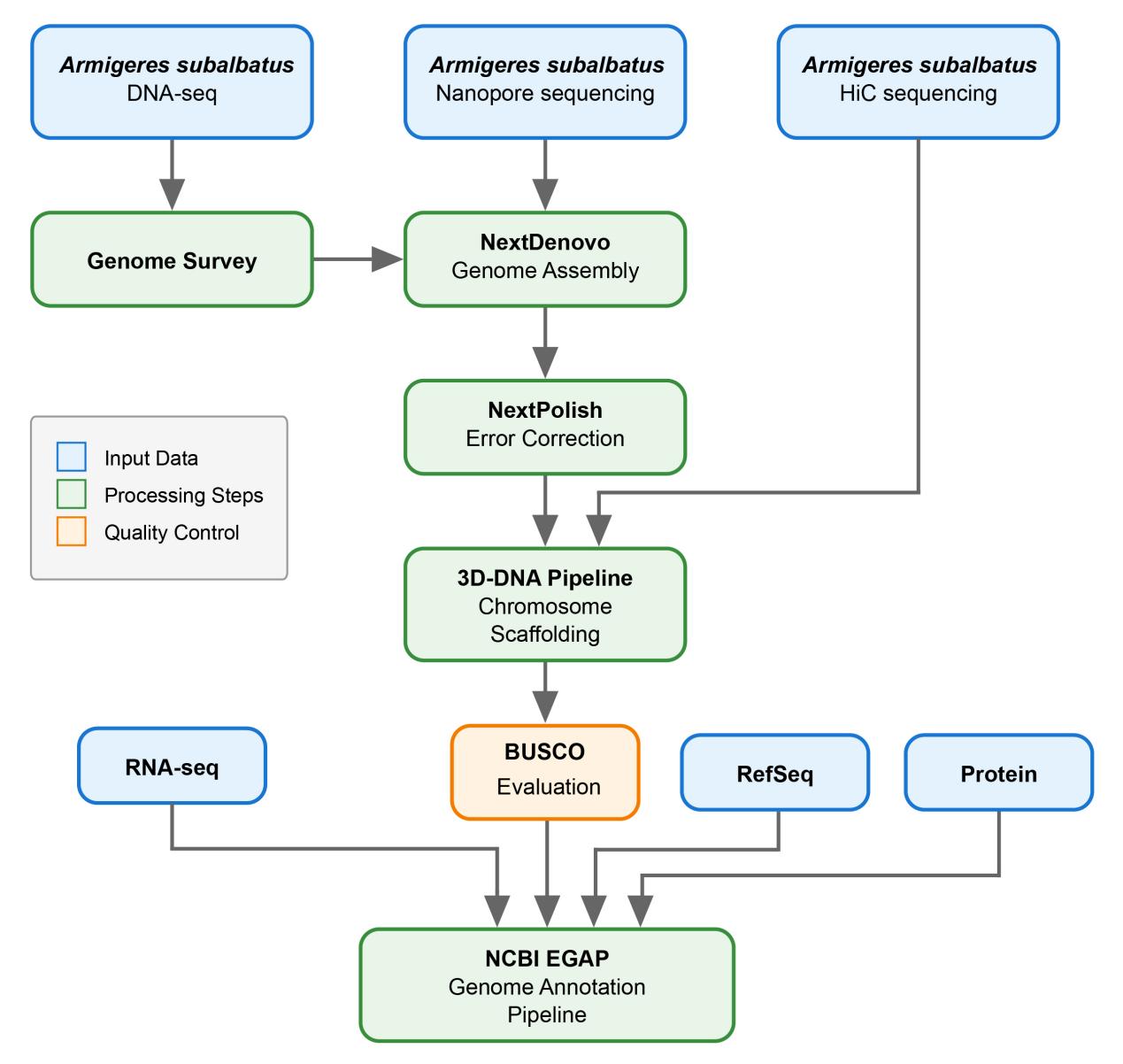


**Figure S1. Pipeline for de novo genome assembly and annotation of *Armigeres subalbatus*.** EGAP, Eukaryotic Genome Annotation Pipeline.


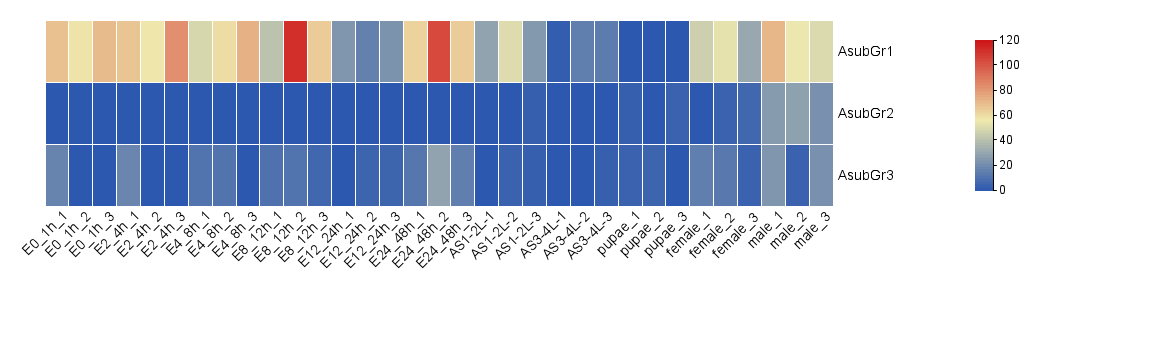


**Figure S2. Carbon dioxide receptors expressed in different stages of *Ar. subalbatus*.**


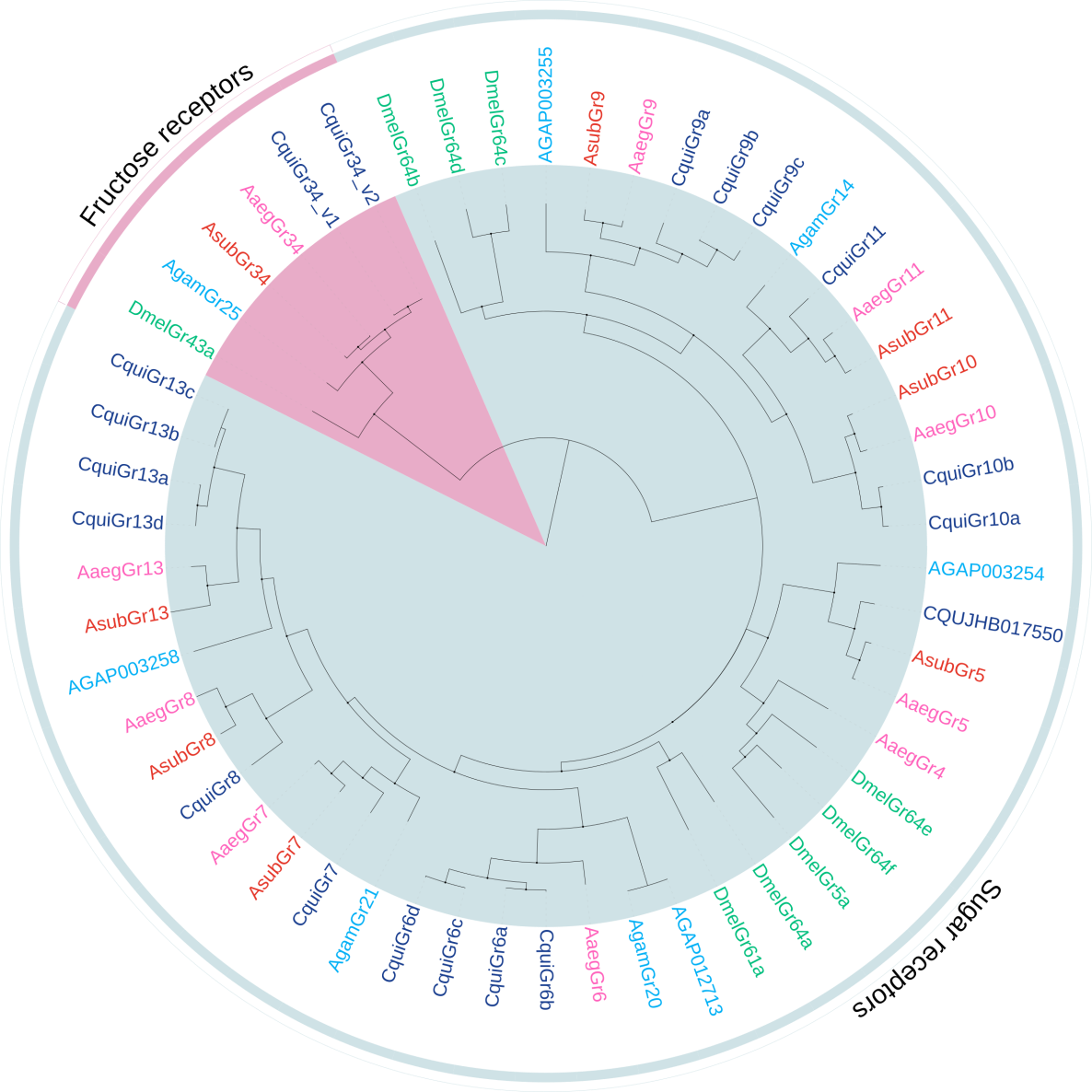


**Figure S3. Phylogenetic relationship of sweet receptors in *D. melanogaster, An. gambae, Ae. aegypti, Cx. quinquefascitus, and Ar. subalbatus*.**


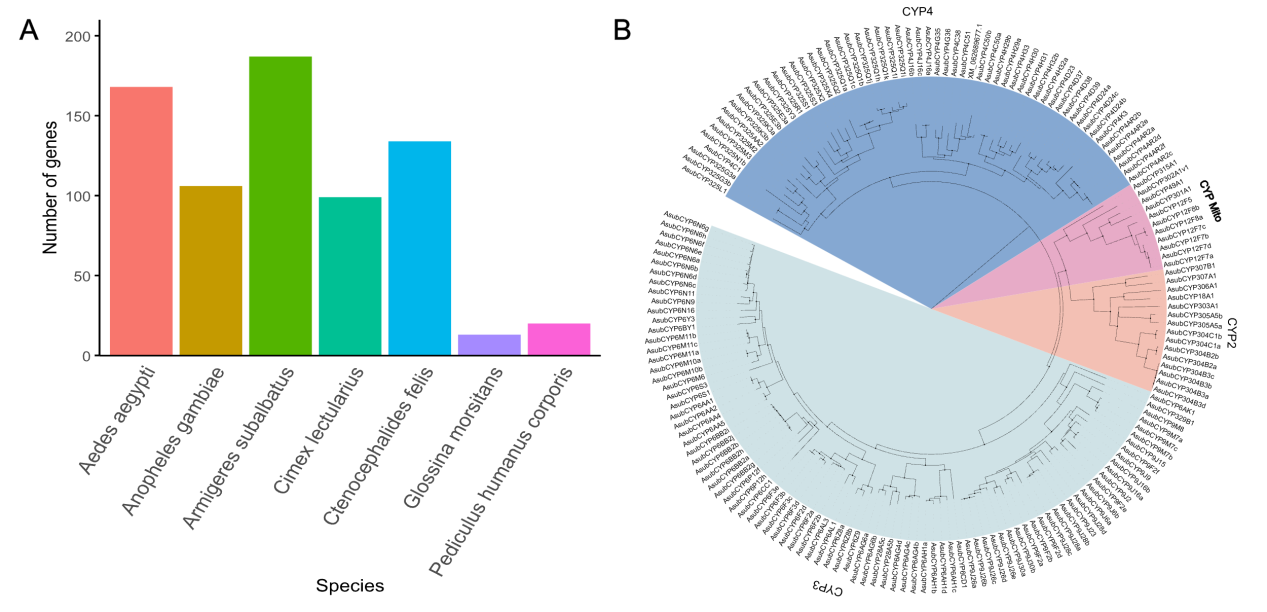


**Figure S4.** (A) Phylogenetic relationship of P450s in *D. melanogaster*, *An. gambae*, *Ae. aegypti*, *Cx. quinquefascitus*, and *A. subalbatus*. (B) Phylogenetic relationship of P450s in *Ar. subalbatus*. They formed four clans (CYP2/3/4/Mito). Clan 3 contained CYP 6/9 is the most expanded P450 family. Clan 3/4 are related to detoxification functions.


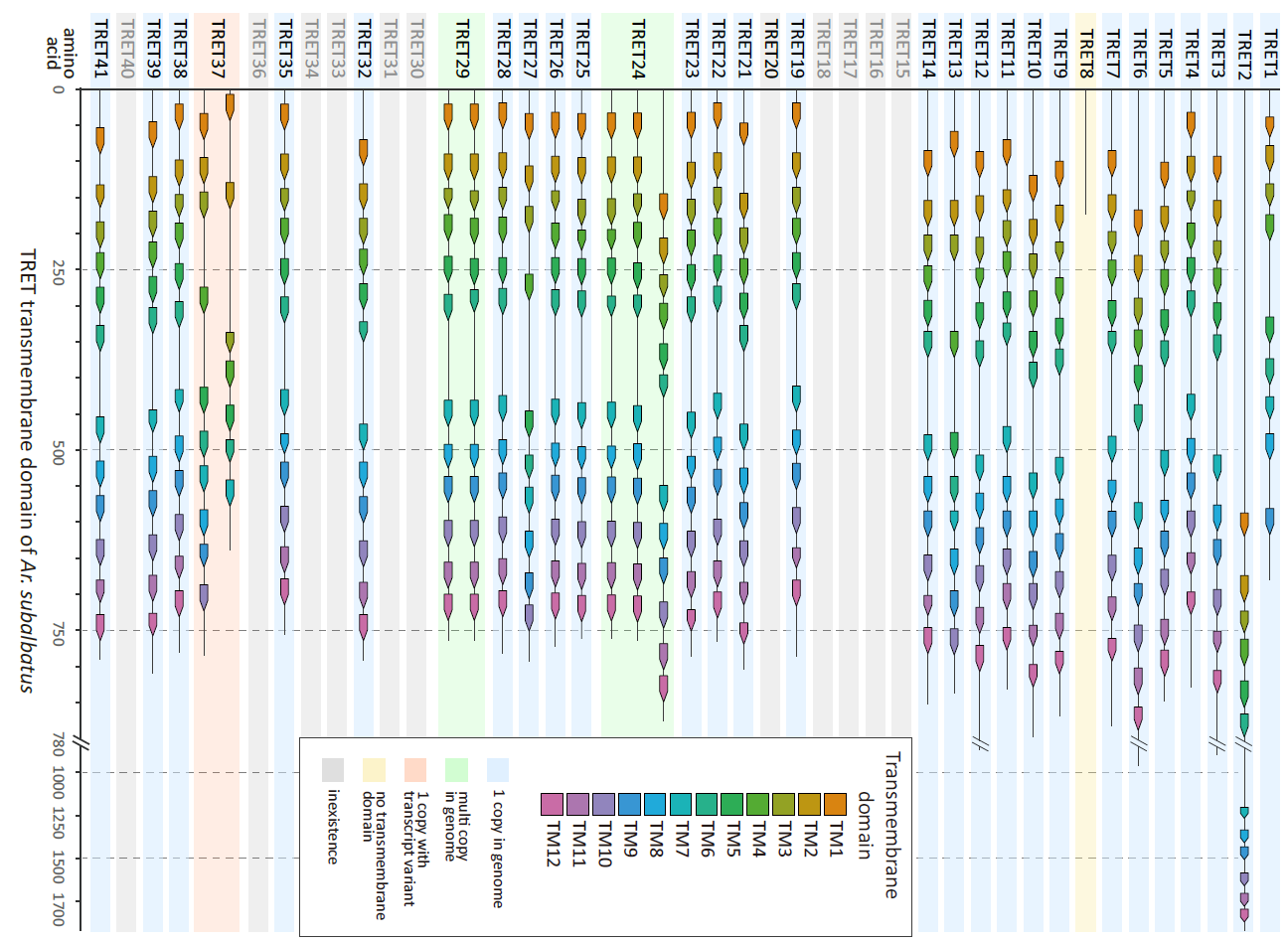


**Figure S5.** **TRET variants exhibiting varying numbers of transmembrane domains in *Ar. subalbatus****.* Variants depicted with a light blue background possess a single copy within the *Ar. subalbatus* genome, whereas those shaded green indicate multiple copies. The TRET37 variant, shaded in lightsalmon, encompasses two isoforms, each containing 7 and 10 transmembrane domains, respectively. Notably, TRET8 (shaded in pale yellow) is unique as it lacks transmembrane domains and boasts the shortest protein sequence among all variants. Conversely, gray shading signifies the absence of corresponding TRET variants in *Ar. subalbatus.*


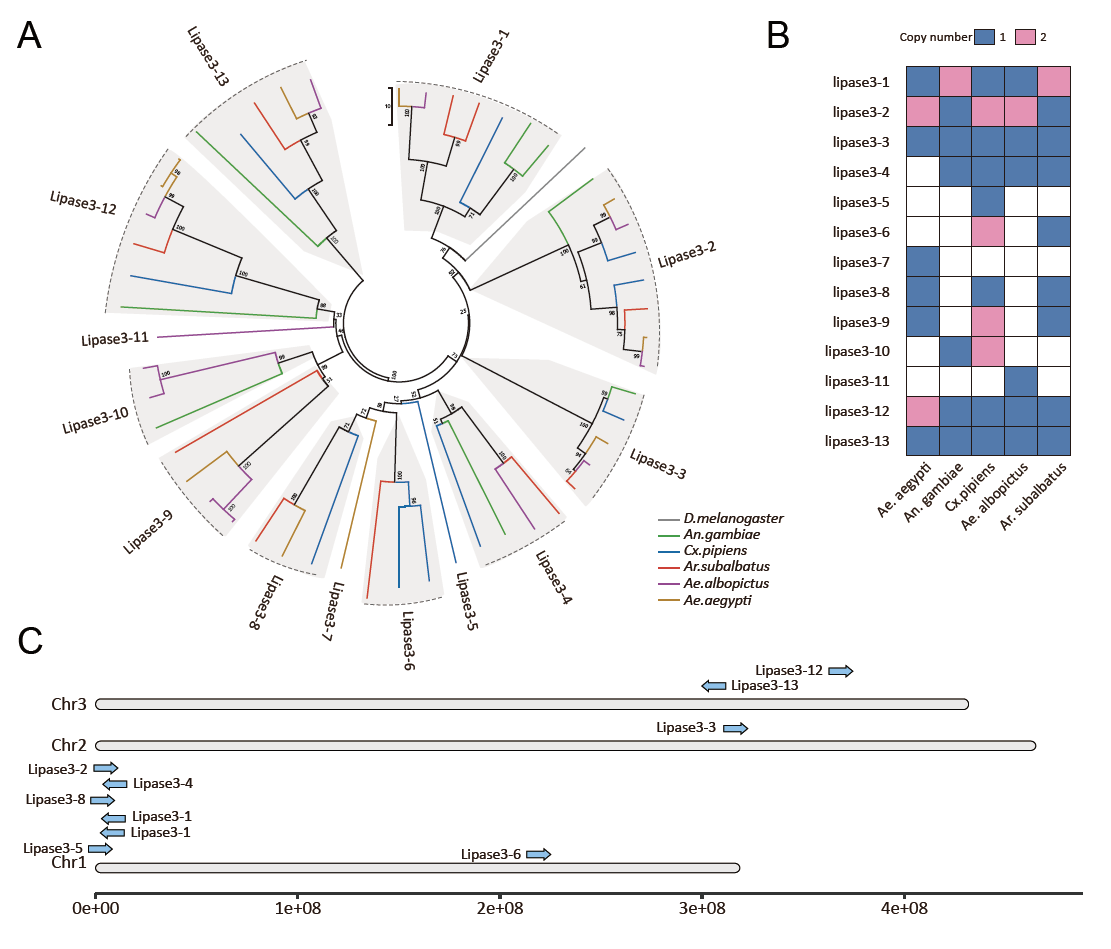


**Figure S6.** **Phylogenetic analysis of *lipase3*.** (A) Thirteen variants of Lipase3 were classified from the aforementioned six insect species. (B) The distribution of Lipase3 variants across the five mosquito species was assessed. (C) The different lipase3 variants located in *Ar. Subalbatus* genome.


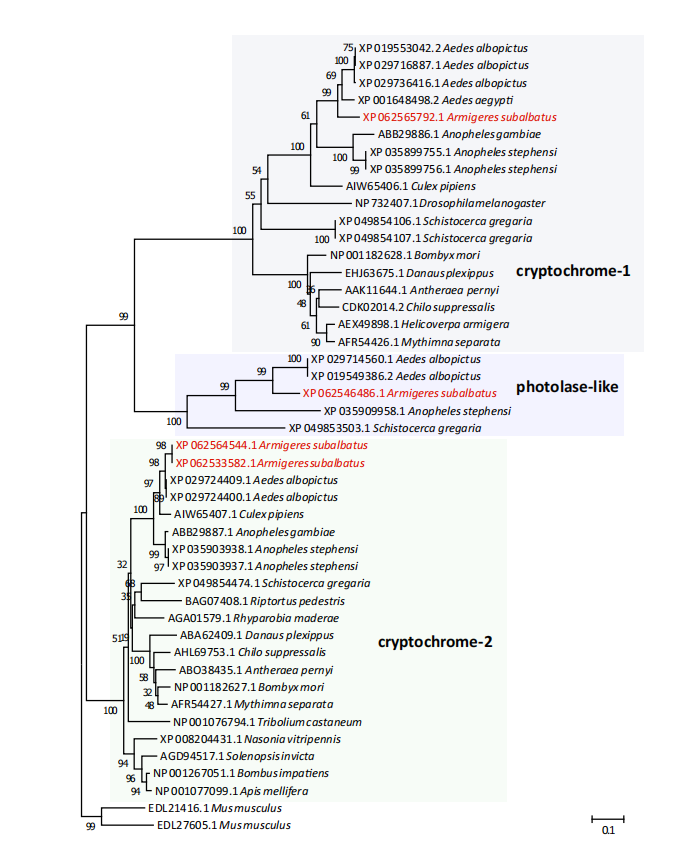


**Figure S7.** **Phylogenetic analysis of the circadian clock gene *cry* (crypyochrome).** The circadain clock gene system of *Ar.subalbatus* is similar to that of *D. plexippus* , specifically in the presence of the *cry*1 and *cry*2 genes.


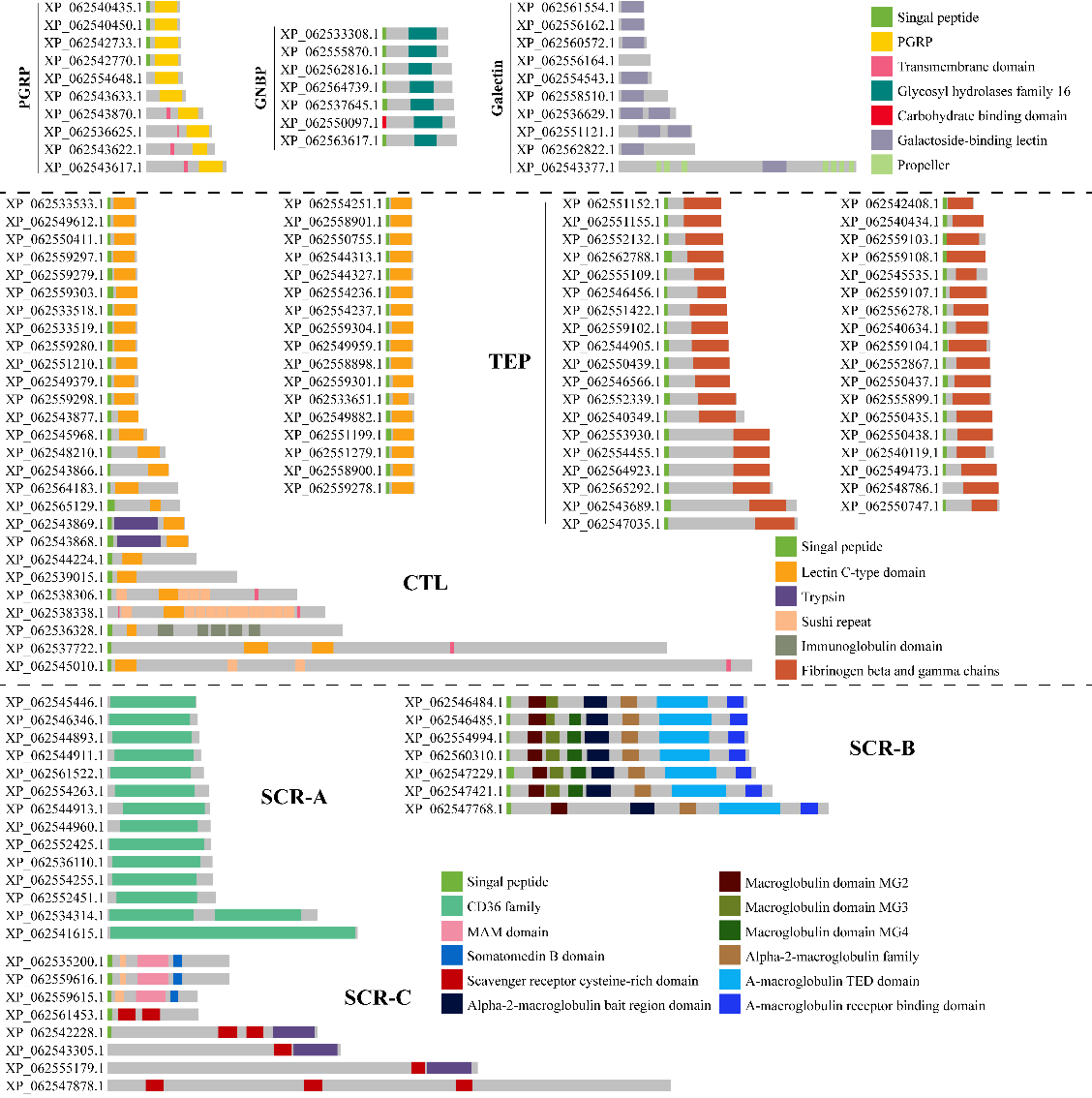


**Figure S8. Domains of recognition proteins.**


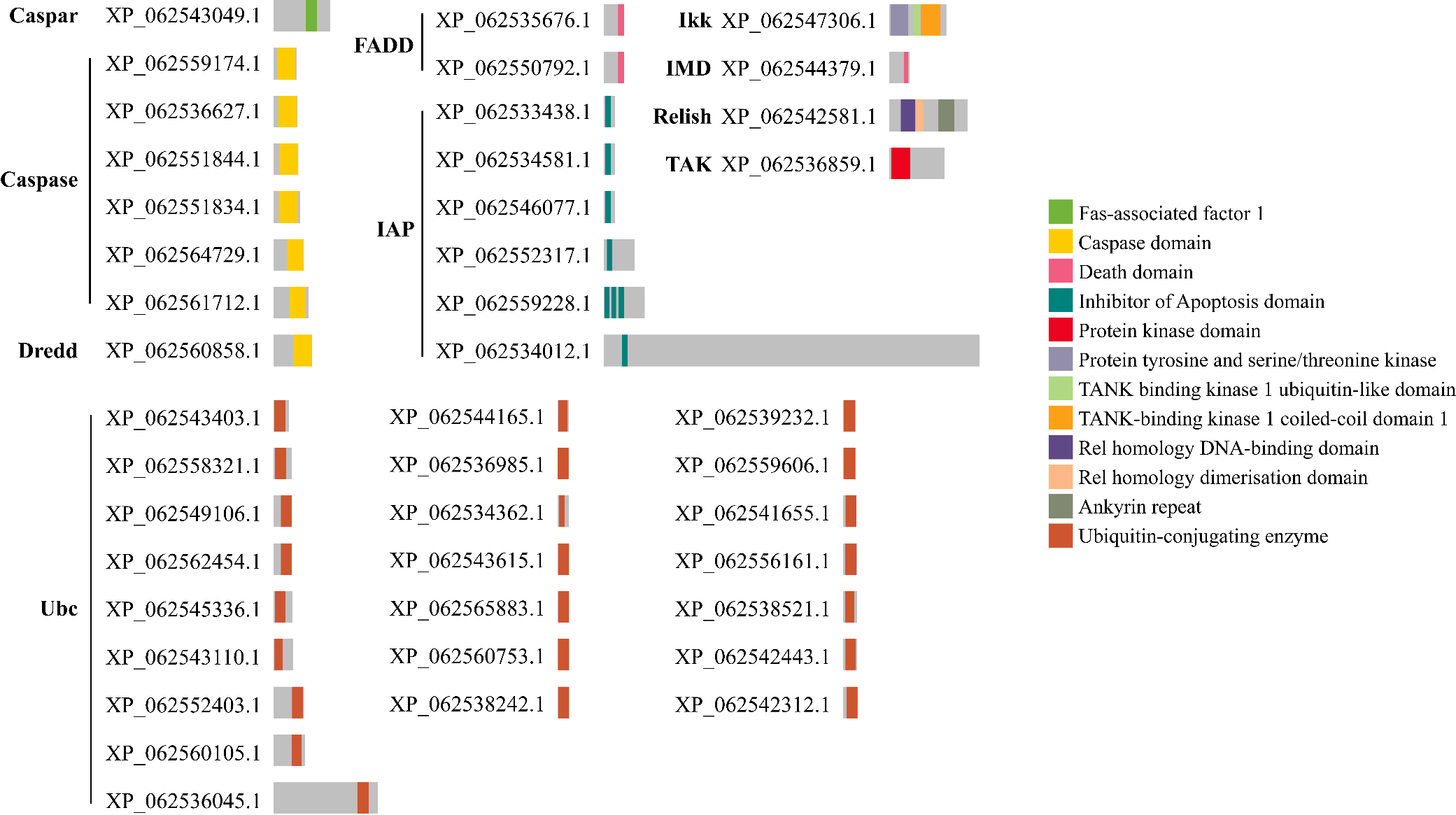


**Figure S9. Domains of proteins in IMD pathway.**


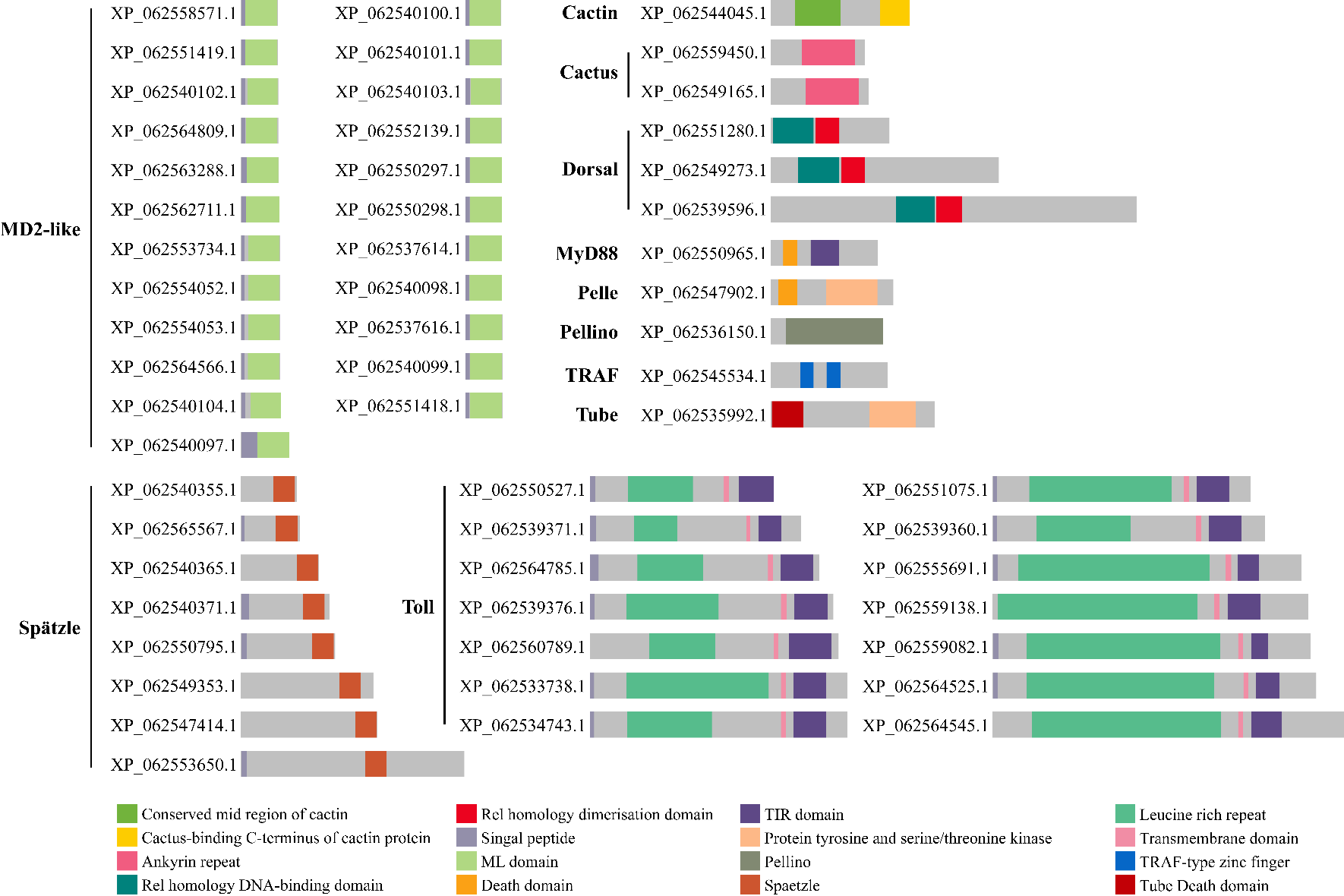


**Figure S10. Domains of proteins in Toll pathway**.


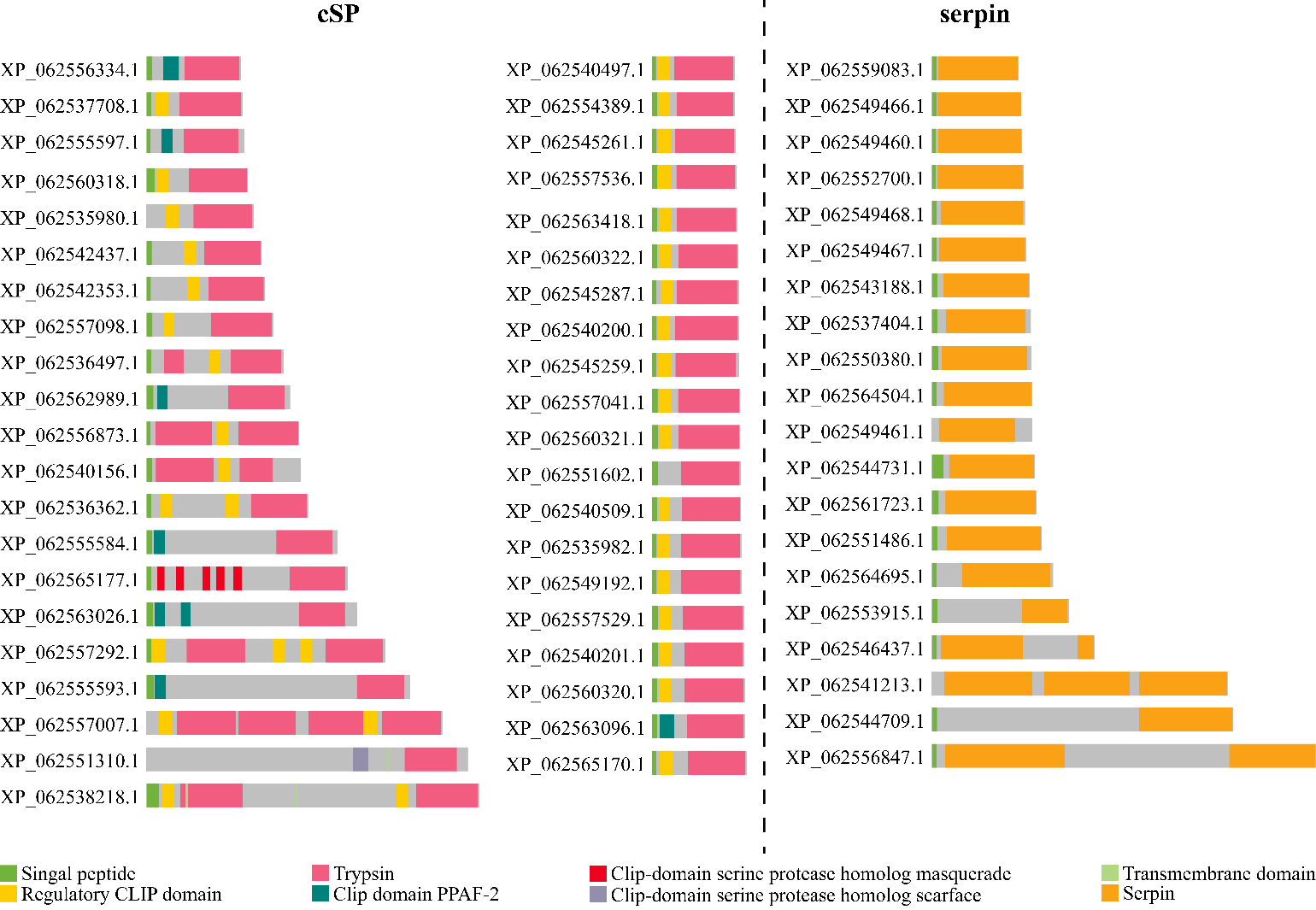


**Figure S11. Domains of CLIPs and serpin.**
